# Hypoxia-induced long non-coding RNA *Malat1* is dispensable for renal ischemia/reperfusion-injury

**DOI:** 10.1038/s41598-018-21720-3

**Published:** 2018-02-21

**Authors:** Malte Kölling, Celina Genschel, Tamas Kaucsar, Anika Hübner, Song Rong, Roland Schmitt, Inga Sörensen-Zender, George Haddad, Andreas Kistler, Harald Seeger, Jan T. Kielstein, Danilo Fliser, Hermann Haller, Rudolf Wüthrich, Martin Zörnig, Thomas Thum, Johan Lorenzen

**Affiliations:** 1 Department of Nephrology, University Hospital, Zürich, Switzerland; 2 Institute of Molecular and Translational Therapeutic Strategies (IMTTS), Hannover Medical School, Hannover, Germany; 3 Department of Nephrology, Hannover Medical School, Hannover, Germany; 4 National Heart and Lung Institute, Imperial College London, London, UK; 5 Semmelweis University, Budapest, Hungary; 6 Georg-Speyer-Haus, Institute for Tumor Biology and Experimental Therapy, Frankfurt, Germany; 7 Department of Nephrology, Städtisches Klinikum Braunschweig GmbH, Braunschweig, Germany; 8 Saarland University Medical Centre, Homburg/Saar, Germany; 9 Department of Internal Medicine, Cantonal Hospital Frauenfeld, Frauenfeld, Switzerland; 10 Excellence Cluster REBIRTH, Hannover Medical School, Hannover, Germany

## Abstract

Renal ischemia-reperfusion (I/R) injury is a major cause of acute kidney injury (AKI). Non-coding RNAs are crucially involved in its pathophysiology. We identified hypoxia-induced long non-coding RNA *Malat1* (Metastasis Associated Lung Adenocarcinoma Transcript 1) to be upregulated in renal I/R injury. We here elucidated the functional role of *Malat1 in vitro* and its potential contribution to kidney injury *in vivo*. *Malat1* was upregulated in kidney biopsies and plasma of patients with AKI, in murine hypoxic kidney tissue as well as in cultured and *ex vivo* sorted hypoxic endothelial cells and tubular epithelial cells. *Malat1* was transcriptionally activated by hypoxia-inducible factor 1-α. *In vitro*, *Malat1* inhibition reduced proliferation and the number of endothelial cells in the S-phase of the cell cycle. *In vivo*, *Malat1* knockout and wildtype mice showed similar degrees of outer medullary tubular epithelial injury, proliferation, capillary rarefaction, inflammation and fibrosis, survival and kidney function. Small-RNA sequencing and whole genome expression analysis revealed only minor changes between ischemic *Malat1* knockout and wildtype mice. Contrary to previous studies, which suggested a prominent role of *Malat1* in the induction of disease, we did not confirm an *in vivo* role of *Malat1* concerning renal I/R-injury.

## Introduction

Ischemia-reperfusion (I/R) injury of the kidney is a major cause of acute kidney injury. Due to its high morbidity and mortality it represents a major socioeconomic health problem^1^. A variety of injurious insults in native kidneys may promote its development (e.g. during cardiac surgery). In addition, it is an unavoidable phenomenon during the kidney transplantation procedure^2^. We and others have previously shown that non-coding RNAs may contribute to the induction or resolution of this process^2^.

The number of RNA transcripts without protein-coding potential exceeds 98% of the human genome^3^. By arbitration these non-coding RNAs (ncRNAs) are divided into long ncRNAs (lncRNAs, ≥200 nucleotides) and small ncRNAs (≤200 nucleotides). Small RNAs such as microRNAs have previously been well characterized. Similar to microRNAs, lncRNAs have recently been shown to regulate gene expression^4^. The study of the function of lncRNAs is still in its infancy and currently a matter of intense research initiatives. LncRNAs may interact with all components of the cellular machinery, including protein, DNA and RNA^4^. The lncRNA metastasis-associated lung adenocarcinoma transcript 1 (*Malat1*) (alternative nomenclature: Nuclear enriched abundant transcript 2 (*Neat2*)) is nuclear enriched and controls alternative splicing by interacting with serine/arginine splicing factors in nuclear speckle domains^5^. It has been shown to regulate hyperglycemia induced inflammatory processes in endothelial cells^6^. It also regulates cell motility via the transcriptional and/or post-transcriptional regulation of motility-related genes^7^. It has been shown to regulate endothelial cell function and vessel growth^8^. In addition, it was recently demonstrated to be induced in kidneys of hypoxic mice, where it was mainly enriched in hypoxic proximal tubular epithelial cells^9^. Moreover, a major initial study found *Malat1* to be one of the most highly regulated non-coding transcripts by hypoxia in a breast cancer cell line^10^. To our knowledge, the *in vivo* role and downstream mechanism of *Malat1* in renal I/R-injury has never been investigated. In the present study *Malat1* was found to be highly induced in ischemic kidneys of mice and humans as well as plasma samples of patients with AKI. *Malat1* was demonstrated to be enriched in sorted proximal tubular epithelial and endothelial cells *ex vivo* following murine renal I/R-injury. We investigated the effects of *Malat1* modulation on dysregulation of signalling pathways as well as kidney function and survival by subjecting *Malat1* knockout mice to renal I/R-injury. Moreover, the downstream mechanisms were analysed in proximal tubular epithelial and endothelial cells. Even though previous studies have suggested a prominent role of *Malat1* in the induction of disease, we did not confirm an effect of *Malat1* loss on the progression of renal I/R-injury.

## Results

### LncRNA *Malat1* is increased in hypoxic/ischemic kidney injury in humans, mice and cells

**Figure 1:**
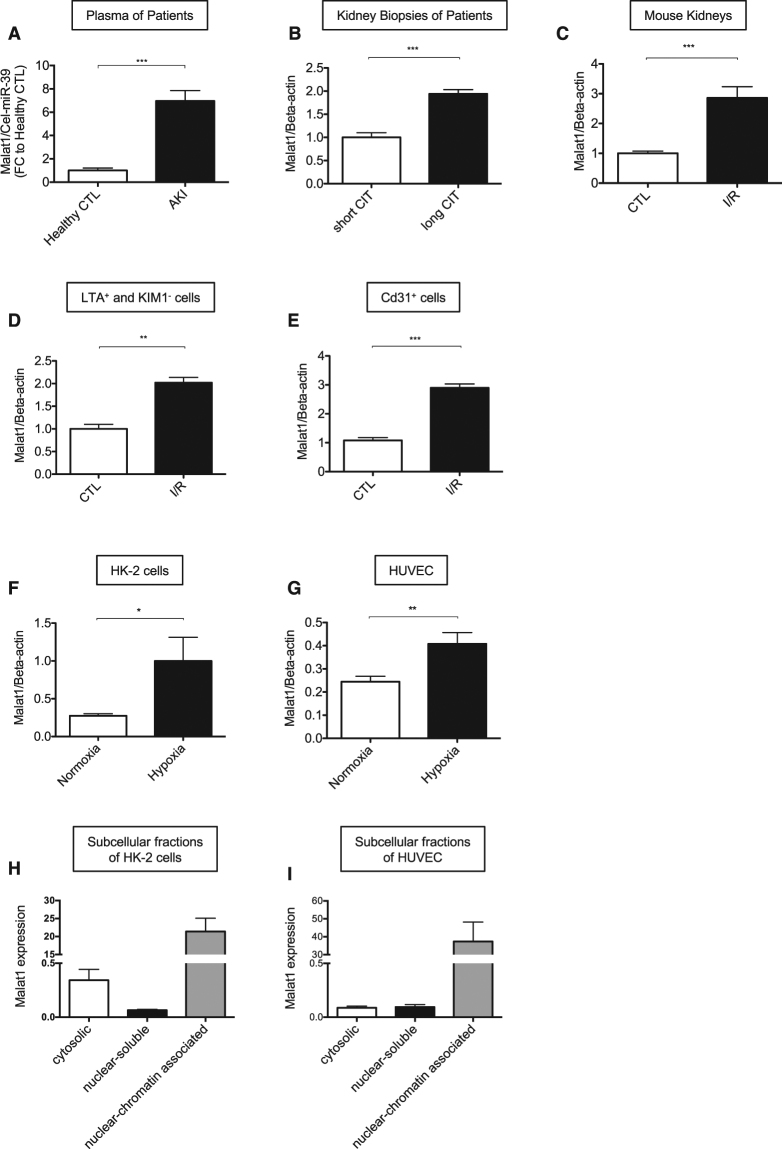
LncRNA *Malat1* is increased in hypoxic/ischemic kidney injury in humans, mice and cells. *Malat1* expression in plasma samples of patients with acute kidney injury (**A**). *Malat1* expression in human kidney biopsies with short- versus long cold ischemia time (CIT) (n = 5) (**B**). *Malat1* expression in mouse kidney following I/R-injury (n = 6) (**C**). *Malat1* expression in sorted LTA^+^ and KIM1^−^ cells of murine kidney after I/R injury (n = 5) (**D**). *Malat1* expression in sorted Cd31^+^ cells of murine kidney after I/R injury (n = 5) (**E**). *Malat1* expression in cultured HK-2 cells (**F**) and HUVEC (**G**) (n = 5 each). *Malat1* expression in subcellular fractions (cytosolic, nuclear soluble, nuclear chromatin associated) of HK-2 cells (**H**) and HUVECs (**I**) (n = 4). *P < 0.05; **P < 0.01; ***P < 0.001; ****P < 0.0001. CIT = cold ischemia time, CTL = control, I/R = ischmemia/reperfusion-injury.

We first assessed the concentration of *Malat1* in plasma samples of patients with acute kidney injury (AKI) and found it to be highly increased compared to healthy controls (Fig. 1A). In addition, the expression of *Malat1* in kidney biopsies of transplant patients with long compared to short cold ischemia time was strongly increased (Fig. 1B). Patient characteristics are shown in Table 1 and Table 2. Moreover, *Malat1* was highly enriched in hypoxic kidneys of mice following induction of I/R-injury (30 minutes of ischemia, 24 h of reperfusion; Fig. 1C). In order to identify the cellular origin of *Malat1* we isolated LTA^+^/KIM1^−^ proximal tubular epithelial cells and Cd31^+^ endothelial cells by fluorescence-associated cell sorting using specific antibodies and lectins from mouse kidneys following I/R-injury. Intriguingly, *Malat1* was found to be enriched in both cell types (Fig. 1D,E). In line with this finding *Malat1* was found to be induced in cultured HUVECs and HK-2 cells by hypoxia/reoxygenation (Fig. 1F,G). We performed a subcellular fractionation in HUVECs and HK-2 cells and found that *Malat1* is mostly nuclear chromatin-associated in both cell types. However, in HK-2 cells *Malat1* was also partly detected in the cytosolic fraction (Fig. 1H,I).

**Table 1:** Demographic, clinical and laboratory characteristics of patients with acute kidney injury.

	Total	Survivors	Non-survivors	p-value
**Number of patients**	77	49	28	0.7
Male (n; %)	40 (52)	26	14	
Female (n; %)	37 (48)	23	14	
**Discipline of ICU admission**				0.9
Medicine (n; %)	29 (38)	19 (40)	10 (36)	
General surgery (n; %)	20 (26)	13 (27)	7 (25)	
Cardiac surgery (n; %)	28 (36)	17 (35)	11 (39)	
**Age (years)**	49 (39–60)	49 (40–61)	50 (38–60)	0.8
**BMI (kg/m** ^**2**^ **)**	25 (22–28)	25 (22–28)	25 (22–28)	0.6
**Indication for RRT**				
eGFR loss >30%	68	44	24	0.4
Oliguria/anuria	48	30	18	0.9
Metabolic acidosis	7	1	6	0.005^*^
Hyperkalemia	6	2	4	0.1
**Sofa score**	13 (11–16)	12 (10–15)	14 (18–18)	0.05^*^
**RIFLE class**				0.2
Risk (n; %)	5 (6)	2 (4)	3 (11)	
Injury (n; %)	21 (27)	16 (33)	5 (18)	
Failure (n; %)	50 (65)	30 (63)	20 (71)	
**APACHE II score**	29 (25–34)	28 (23–34)	34 (29–28)	0.4
**CRP (mg/L)**	128 (47–202)	96 (48–205)	141 (56–198)	0.5
**MAP (mmHg)**	82 (71–94)	84 (70–97)	78 (73–86)	0.3
**Heart rate (bpm)**	99 (88–109)	92 (83–104)	100 (96–117)	0.6

APACHE II = Acute Physiology and Chronic Health Evaluation II; BMI = body mass index; CRP = C-reactive protein; eGFR = estimated glomerular filtration rate; ICU = intensive care unit; MAP = mean arterial blood pressure; n = number of patients; RRT = renal replacement therapy; SOFA = Sequential Organ Failure Assessment.

**Table 2:** Demographics of transplant patients with long and short cold ischemia time (CIT).

		Short CIT	Long CIT
**Recipient gender**	male / female	1 / 4	2 / 3
**Recipient age**	years	46.2 ± 3.9	47.0 ± 4.7
**Cause of ESRD**			
Glomerulonephritis	n	3	2
Hypertensive/diabetic nephropathy	n	1	2
unknown	n	1	1
**Cold ischemia time (minutes)**		208 ± 56	707 ± 73

### *Malat1* expression in HUVEC and HK-2 cells is transcriptionally activated by Hypoxia-inducible Factor-1α

**Figure 2:**
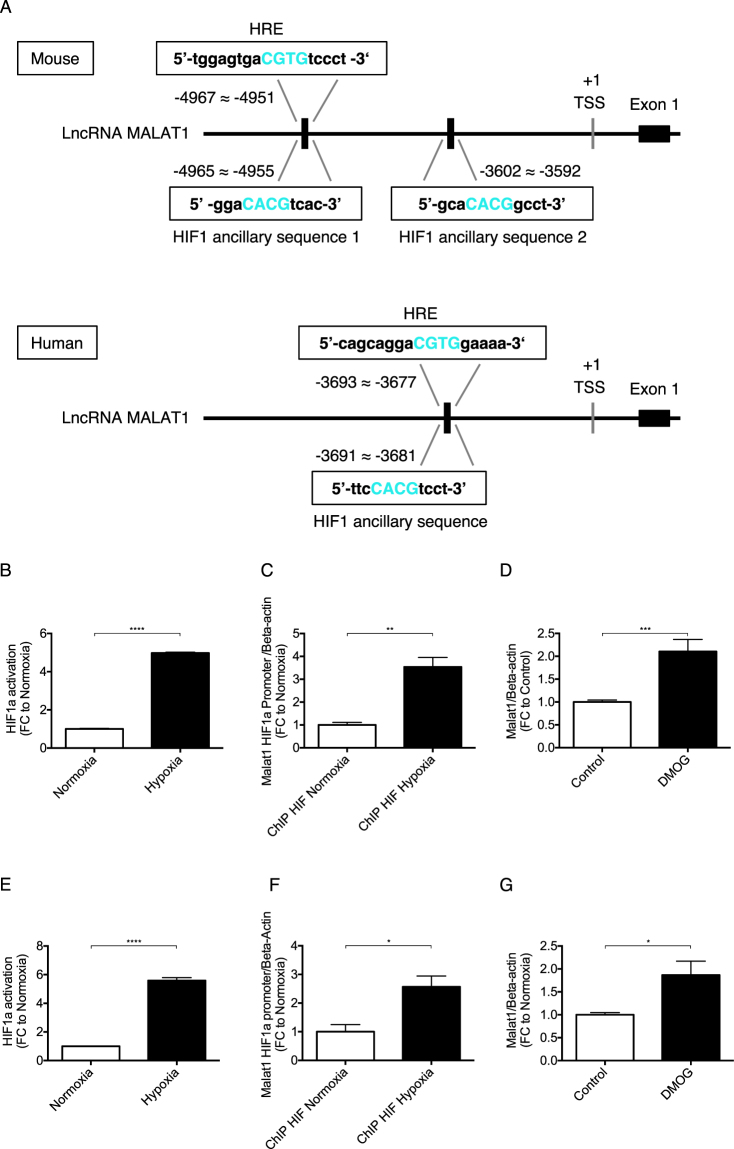
*Malat1* gene expression in HUVECs and HK-2 cells is transcriptionally activated by HIF-1α (=HIF1a). Upstream promoter region of *Malat1* spanning 5 kb from the transcriptional start site (TSS) identifies 1 putative Hypoxia Response Elements (HRE) and 2 HIF1 ancillary sequence in mice as well as 1 putative Hypoxia Response Elements (HRE) and 1 HIF1 ancillary sequence in humans (**A**). Transcriptional activation of HIF-1α in hypoxic HUVECs (**B**) and HK-2 cells (**E**) detected by transcriptional activation ELISA (n = 4). qPCR of *Malat1* primer amplifying the HIF-1α– promoter region after immunoprecipitation of hypoxic HUVECs (**C**) and HK-2 cells (**F**) (n = 5). *Malat1* expression after Dimethyloxaloylglycin (DMOG) treatment of HUVECs (**D**) and HK-2 cells (**G**) for 24 h. *P < 0.05; **P < 0.01; ***P < 0.001; ****P < 0.0001. ChIP = Chromatin immunoprecipitation, HIF-1α = Hypoxia-inducible factor 1 alpha, TSS = Transcriptional Start Site.

To identify transcription factors activating *Malat1* in response to hypoxia, we employed the Genomatix suite of sequence analysis tool (MatInspector). One putative hypoxia response elements (HRE) and 2 Hypoxia-inducible Factor 1 (HIF1) ancillary sequence were found within the promoter region of the *Malat1* gene 5 kb upstream of the transcriptional start site in mice as well as one putative HRE and 1 Hypoxia-inducible Factor 1 (HIF1) ancillary sequence in humans (Fig. 2A). In an ELISA based transcriptional activation study, we found Hypoxia-inducible Factor 1 alpha (HIF-1α) highly induced in HUVECs (Fig. 2B) and HK-2 cells (Fig. 2E) upon exposure to hypoxia, as expected. Immunoblotting confirmed HIF-1α activation by hypoxia (Supplemental Fig. 1A) as well as transcription of established HIF-1 target genes CA9, Glut1, PHD2 in HK-2 cells (Supplemental Fig. 1B–D). We used a specific HIF-1α antibody to chromatin-immunoprecipitate HIF-1α bound to the promoter region of *Malat1*. Subsequently, we designed specific primers for quantitative PCR analysis detecting the HIF-1α binding region in the *Malat1* promoter region and found a significantly increased expression level in HUVECs (Fig. 2C) and HK-2 cells (Fig. 2F). In order to stabilize HIF-1α under normoxic conditions, we treated HUVECs and HK-2 cells with Dimethyloxaloylglycine (DMOG), an inhibitor of the prolyl hydroxylase (PHD) and the asparaginyl hydroxylase factor inhibiting HIF (FIH). DMOG induced activation of HIF-1α increased *Malat1* expression in HUVECs (Fig. 2D) and HK-2 cells (Fig. 2G). Altogether, we identified HIF-1α as transcriptional activator of *Malat1* in HUVECs and HK-2 cells. HIF-1α is part of a heterodimer with HIF-β activating hypoxia-sensitive transcription^11^.

### Functional role of *Malat1* in HUVEC and HK-2 cell biology

**Figure 3:**
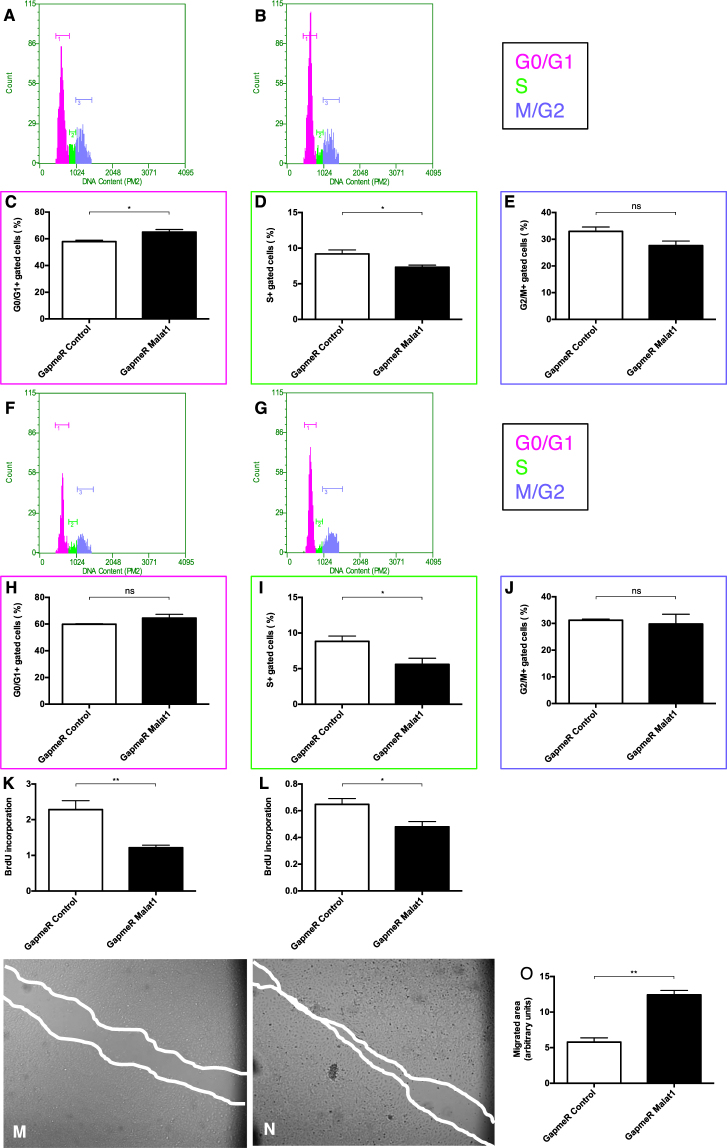
Functional role of *Malat1* in HUVEC biology. DNA histograms showing HUVECs detected in G0/G1, S, or G2/M phase of the cell cycle after GapmeR Control (**A**) and GapmeR *Malat1* (**B**) treatment under basal conditions. Quantification of HUVECs detected in G0/G1 phase (**C**), S phase (**D**), and G2/M phase (**E**) under basal conditions (n = 5). DNA histograms showing HUVECs detected in G0/G1, S, or G2/M phase of the cell cycle after GapmeR Control (F) and GapmeR *Malat1* (**G**) treatment after hypoxia/reoxygenation. Quantification of HUVECs detected in G0/G1 phase (H), S phase (**I**), and G2/M phase (**J**) after hypoxia/reoxygenation (n = 5). BrdU incorporation in HUVECs treated with GapmeR Control and GapmeR *Malat1* under normoxic conditions (**K**) and after hypoxia/reoxygenation (**L**) (n = 5). Scratch migration analysis comparing the migrated area of GapmeR Control- (**M**) and GapmeR *Malat1* (**N**) treated HUVEC at 12 hours and quantification of the results (**O**). *P < 0.05; **P < 0.01; ***P < 0.001; ****P < 0.0001. BrdU = Bromodeoxyuridine incorporation.

To investigate the functional role of *Malat1 in vitro*, we used antisense oligonucleotides (GapmeR) to silence *Malat1* expression. First, we analysed the impact of *Malat1* on cell cycle regulation under basal normoxic conditions and in response to hypoxia/reoxygenation. In this context, cellular DNA was labeled with propidium iodide to differentiate cells in all phases of the cell cycle. Under normoxic conditions, *Malat1* antagonism decreased the G1- to S-phase transition in HUVECs (Fig. 3A–E). Likewise, cell cycle progression was diminished in response to hypoxia/reoxygenation in HUVECs demonstrated by a significantly reduced amount of HUVECs entering the S-phase of the cell cycle (Fig. 3F–J). Furthermore, we studied the impact of *Malat1* on the proliferation rate of HUVECs. We observed, that silencing of *Malat1* in HUVECs reduced the proliferation rate significantly in both normoxia and hypoxia/reoxygenation (Fig. 3K,L). In addition, we compared the migratory capacity of HUVECs depending on *Malat1* expression. Intriguingly, antagonizing *Malat1* in HUVECs significantly increased the capacity to migrate (Fig. 3M–O). To reveal an effect of *Malat1* on apoptosis induction in HUVECs, we stained cells with Annexin and 7AAD and subsequently used fluorescent-activated cell sorting to detect cells undergoing early apoptosis (Annexin + /7AAD-). Here we found a protective effect of *Malat1* antagonism on early apoptosis after hypoxia/reoxygenation (Supplemental Fig. 2A). Likewise, *Malat1* inhibition decreased Caspase 3/7 activity in hypoxic/reoxygenated HUVECs (Supplemental Fig. 2B). In contrast, *Malat1* silencing in HK-2 cells did not result in any functional alteration, concerning cell cycle regulation in normoxia (Supplemental Fig. 3A–C) and upon hypoxia/reoxygenation (Supplemental Fig. 3D–F), proliferation rate in normoxia and upon hypoxia/reoxygenation (Supplemental Fig. 3G,H) as well as apoptosis induction upon hypoxia/reoxygenation (Supplemental Fig. 3I–J). These findings suggest *Malat1* to impact on endothelial cell cycle progression, proliferation, migration capacity and apoptosis induction, at least *in vitro*.

### *In vivo* role of *Malat1* in tubular epithelial cell injury, proliferation and capillary density

**Figure 4:**
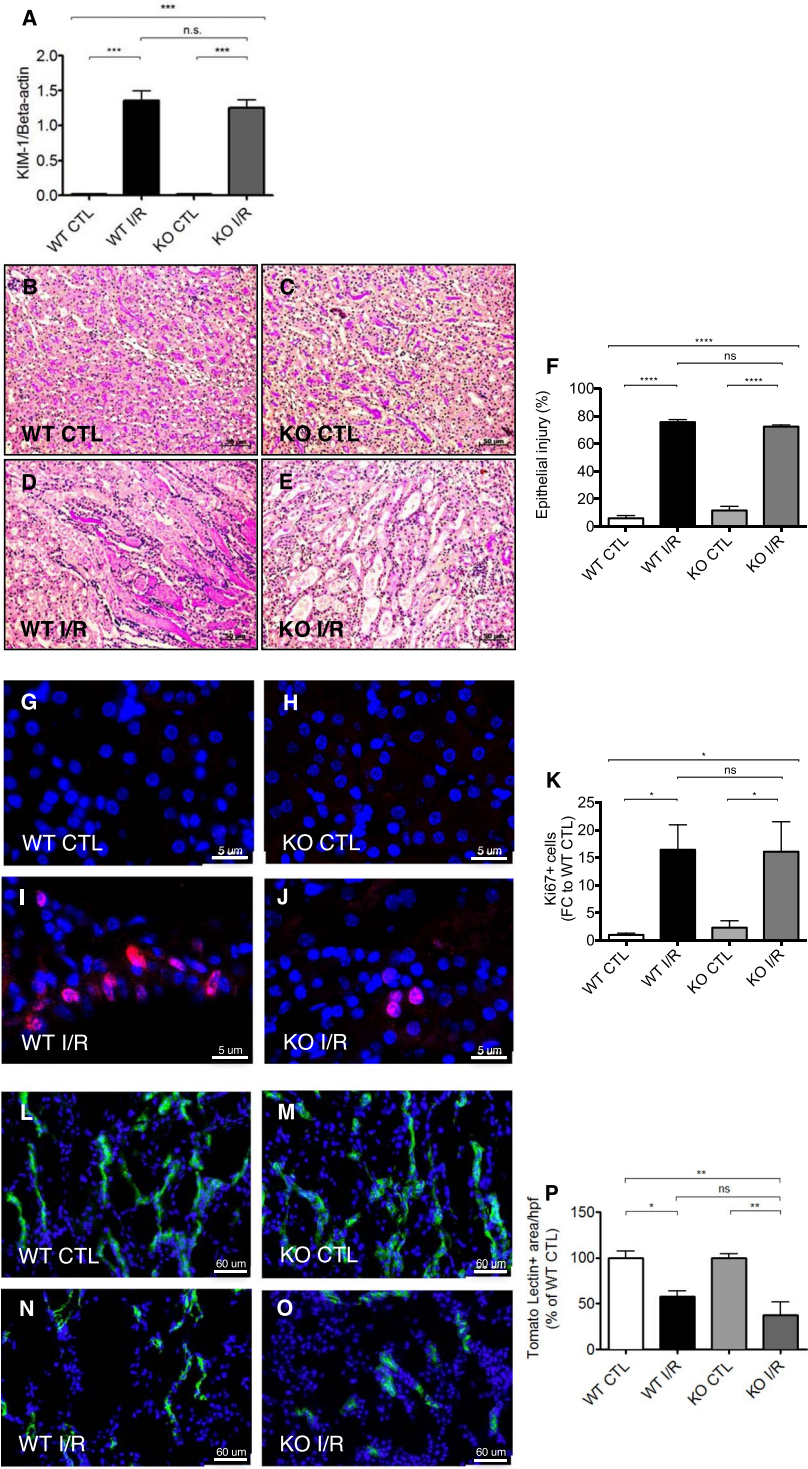
*In vivo* role of *Malat1* in tubular epithelial cell injury, proliferation and capillary density. KIM-1 gene expression after 24 h of I/R injury (**A**). Epithelial injury analyzed by PAS staining in wild-type control mice (**B**), knockout control mice **(C**), wildtype mice with ischemia/reperfusion injury (**D**) and knockout mice with ischemia/reperfusion injury (**E**) and quantification of the results (**F**). Detection of proliferating cells by Ki67 staining after 24 h of reperfusion in wildtype control mice (**G**), knockout control mice (**H**), wildtype mice with ischemia/reperfusion injury (**I**) and knockout mice with ischemia/reperfusion injury (**J**) and quantification of the results (**K**). Evaluation of capillary density by Tomato-Lectin staining after 24 h of reperfusion in wildtype control mice (**L**), knockout control mice (**M**), wildtype mice with ischemia/reperfusion injury (**N**) and knockout mice with ischemia/reperfusion injury (**O**) and quantification of the results (**P**). *P < 0.05; **P < 0.01; ***P < 0.001; ****P < 0.0001; n = 6 mice in each group. KIM-1 = kidney injury molecule-1; KO = knockout, WT = wildtype, CTL = control, I/R = ischemia/reperfusion-injury, FC = foldchange.

As we found *Malat1* upregulated in patients and mice upon renal I/R Injury (Fig. 1), our aim was to elucidate the contribution of *Malat1* to renal I/R injury *in vivo*. We used *Malat1* knockout - and wildtype mice on a C57BL/6 background and induced unilateral renal I/R injury to analyze possible effects on epithelial- and endothelial injury. First, we detected increased renal expression levels of Kidney Injury Molecule – 1 (KIM-1) after I/R injury in wildtype mice, that were increased similarly in mice lacking the *Malat1* gene (Fig. 4A). PAS staining was performed to score the degree of outer medullary tubular epithelial injury. After unilateral renal I/R injury, approximately 70–80% of tubules were injured in both *Malat1* knockout and wildtype mice (Fig. 4B–F). To detect proliferating cells, we performed Ki67 staining and observed proliferating cells in *Malat1* knockout and wildtype mice to be equally increased (Fig. 4G–K). Capillary integrity upon I/R injury was analyzed by Tomato-Lectin staining. Severe capillary rarefaction was found in *Malat1* knockout and wildtype mice without any differences (Fig. 4L–P). These results indicate, that *Malat1* is not associated with impaired proliferation or epithelial and capillary integrity in post-ischemic kidneys *in vivo*.

### Inflammatory cell influx upon unilateral renal I/R injury

**Figure 5:**
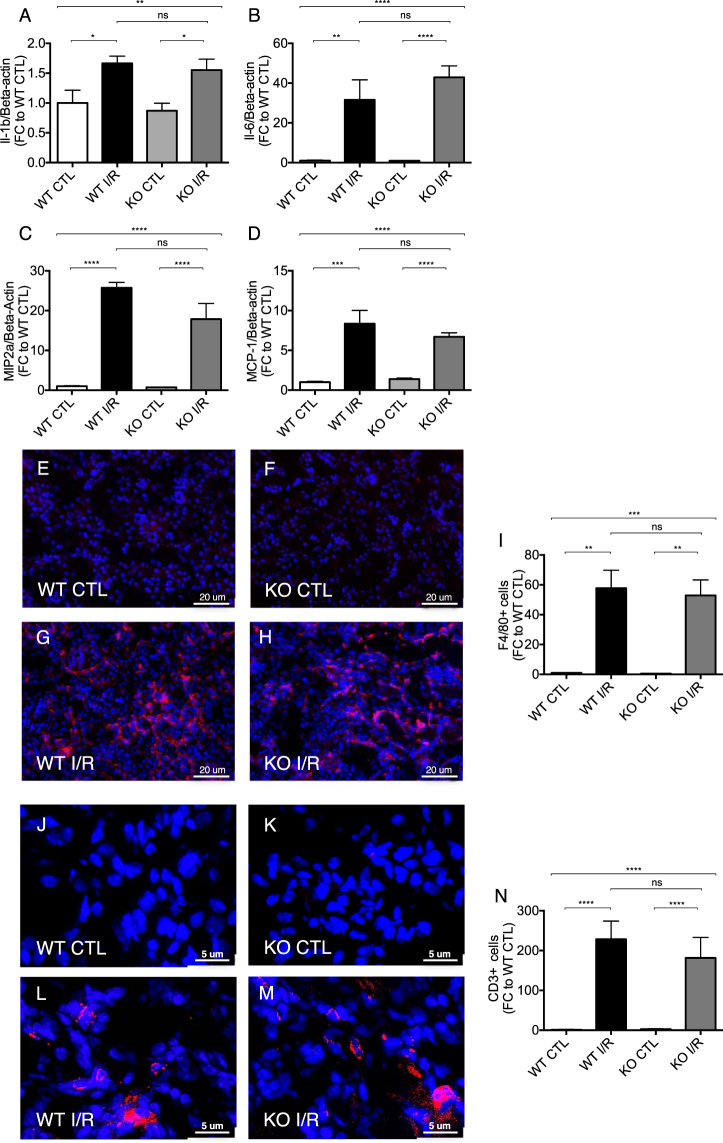
Inflammatory gene expression and cell influx upon unilateral renal I/Rinjury. Il-1b (**A**). MIP2a (**B**), Il-6 (**C**) and MCP-1 expression (**D**) in ischemic murine kidneys. Analysis of F4/80 (red) positive macrophage infiltration in wildtype control (**E**), knockout control (**F**), wildtype with ischemia/reperfusion injury (**G**) and knockout mice with ischemia/reperfusion injury (**H**) as well as quantification of the results (**I**) at 7 days of reperfusion. Analysis of CD3 (red) positive T-cell infiltration in wildtype control (**J**), knockout control (**K**), wildtype with ischemia/reperfusion injury (**L**) and knockout mice with ischemia/reperfusion injury (**M**) as well as quantification of results (**N**) at 7 days of reperfusion. *P < 0.05; **P < 0.01; ***P < 0.001; ****P < 0.0001; n = 6 mice in each group. Il-1b = Interleukin-1beta, MIP2a = Macrophage inflammatory protein-2 alpha; Il-6 = Interleukin-6; MCP-1 = monocyte chemoattractant protein – 1, KO = knockout, WT = wildtype, CTL = control, I/R = ischemia/reperfusion-injury, FC = foldchange.

Renal I/R injury is associated with inflammatory cell infiltration. Accordingly, we tested, whether *Malat1* mediates inflammatory responses. Expression levels of Il-1beta (Interleukin-1beta), MIP2a (macrophage inflammatory protein 2 alpha), Il-6 (Interleukin-6) and MCP-1 (monocyte chemoattractant protein-1) were highly upregulated after renal I/R injury, but without any signs of amelioration in mice lacking the *Malat1* gene (Fig. 5A–D). Moreover, F4/80 staining revealed that macrophages were infiltrating the kidney tissue of *Malat1* knockout and wildtype mice upon unilateral renal I/R injury to the same degree (Fig. 5E–I). Likewise, T-cell infiltration (CD3 staining) upon unilateral renal I/R injury was not affected by *Malat1* loss (Fig. 5J–N). Taken together, these data indicate that *Malat1* does not affect renal I/R-injury induced inflammatory cell infiltration.

### Accumulation of interstitial fibrosis after unilateral renal I/R injury

**Figure 6:**
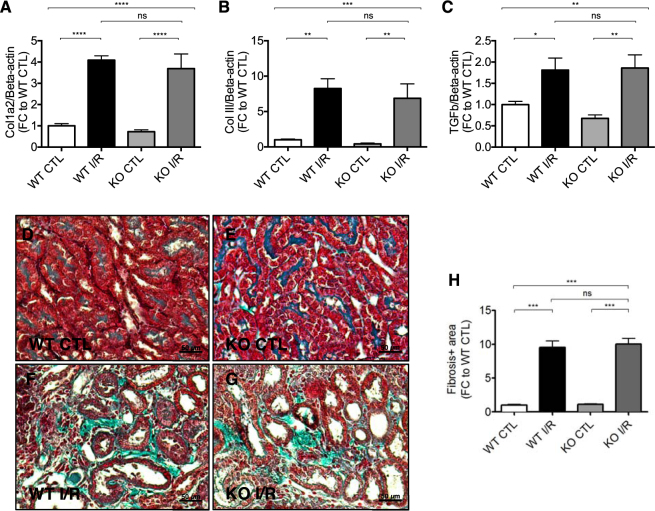
Accumulation of interstitial fibrosis after unilateral renal I/R injury at 7 days of reperfusion. Col1a2 (**A**), Col III (**B**) and TGFb (C) expression in ischemic murine kidneys. Detection of interstitial fibrosis by Massons’s trichrome staining in wildtype control (**D**), knockout control (**E**), wildtype with ischemia/reperfusion injury (**F**) and knockout mice with ischemia/reperfusion injury (**G**) as well as quantification of the results (**H**). *P < 0.05; **P < 0.01; ***P < 0.001; ****P < 0.0001. Col III = Collagen 3, TGFbeta = Transforming growth factor beta, KO = knockout, WT = wildtype, CTL = control, I/R = ischemia/reperfusion-injury, FC = foldchange. n = 6 mice in each group.

In the course of renal I/R injury, accumulation of tubulointerstitial fibrosis ultimately results in an impairment of kidney function. In order to investigate a potential contribution of *Malat1*, we analyzed mRNA levels of pro-fibrotic genes and additionally used Masson’s trichrome staining to detect interstitial fibrosis. Expectedly, Col1a2 (Collagen1-alpha2), Col III (Collagen 3) and TGF-beta (Transforming growth factor beta) expression levels were increased after unilateral renal I/R injury, but there was no significant difference between *Malat1* knockout and wildtype mice (Fig. 6A–C). Interstitial fibrosis was increased after renal I/R injury, but did not show detectable differences between *Malat1* knockout and wildtype mice (Fig. 6D–H).

### Impact of *Malat1* on kidney function and survival after bilateral renal I/R injury

**Figure 7:**
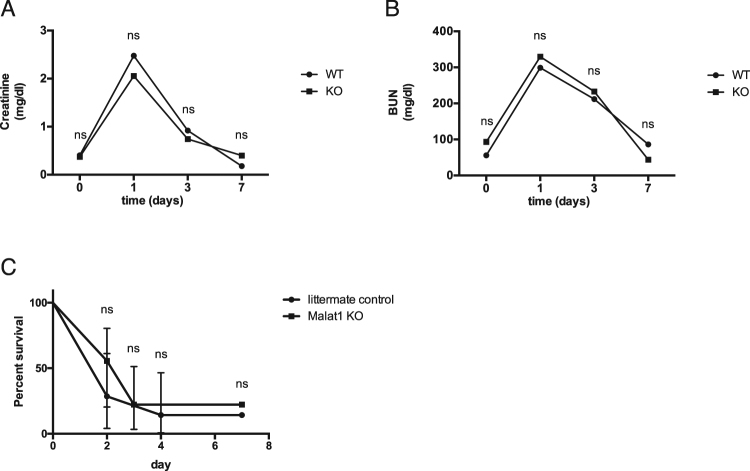
Impact of *Malat1* on kidney function and survival after bilateral renal I/R injury. Creatinine levels in *Malat1* knockout and wildtype mice before induction of ischemia/reperfusion injury, 1 day after ischemia/reperfusion injury, 3 days after ischemia/reperfusion injury and 7 days after ischemia/reperfusion injury (**A**). Urea levels in *Malat1* knockout and wildtype mice before induction of ischemia/reperfusion injury, 1 day after ischemia/reperfusion injury, 3 days after ischemia/reperfusion injury and 7 days after ischemia/reperfusion injury (**B**). Survival study in *Malat1* knockout- and wildtype mice after bilateral renal ischemia/reperfusion injury (**C**). *P < 0.05; **P < 0.01; ***P < 0.001; ****P < 0.0001. KO = knockout, WT = wildtype. n = 10 mice in each group.

To evaluate the influence of *Malat1* on kidney function, we collected blood samples of mice before, at day 1, day 3 and day 7 after bilateral renal I/R injury in *Malat1* knockout and wildtype mice and measured creatinine and urea levels. Creatinine and urea levels in *Malat1* knockout and wildtype mice were similarly increased at day 1 after bilateral renal I/R injury and decreased continuously without any significant difference (Fig. 7A,B). In addition, our survival study of *Malat1* knockout and wildtype mice subjected to bilateral renal I/R injury showed no significant differences (Fig. 7C).

### Whole genome RNA analysis in *Malat1* knockout and wildtype mice

**Figure 8:**
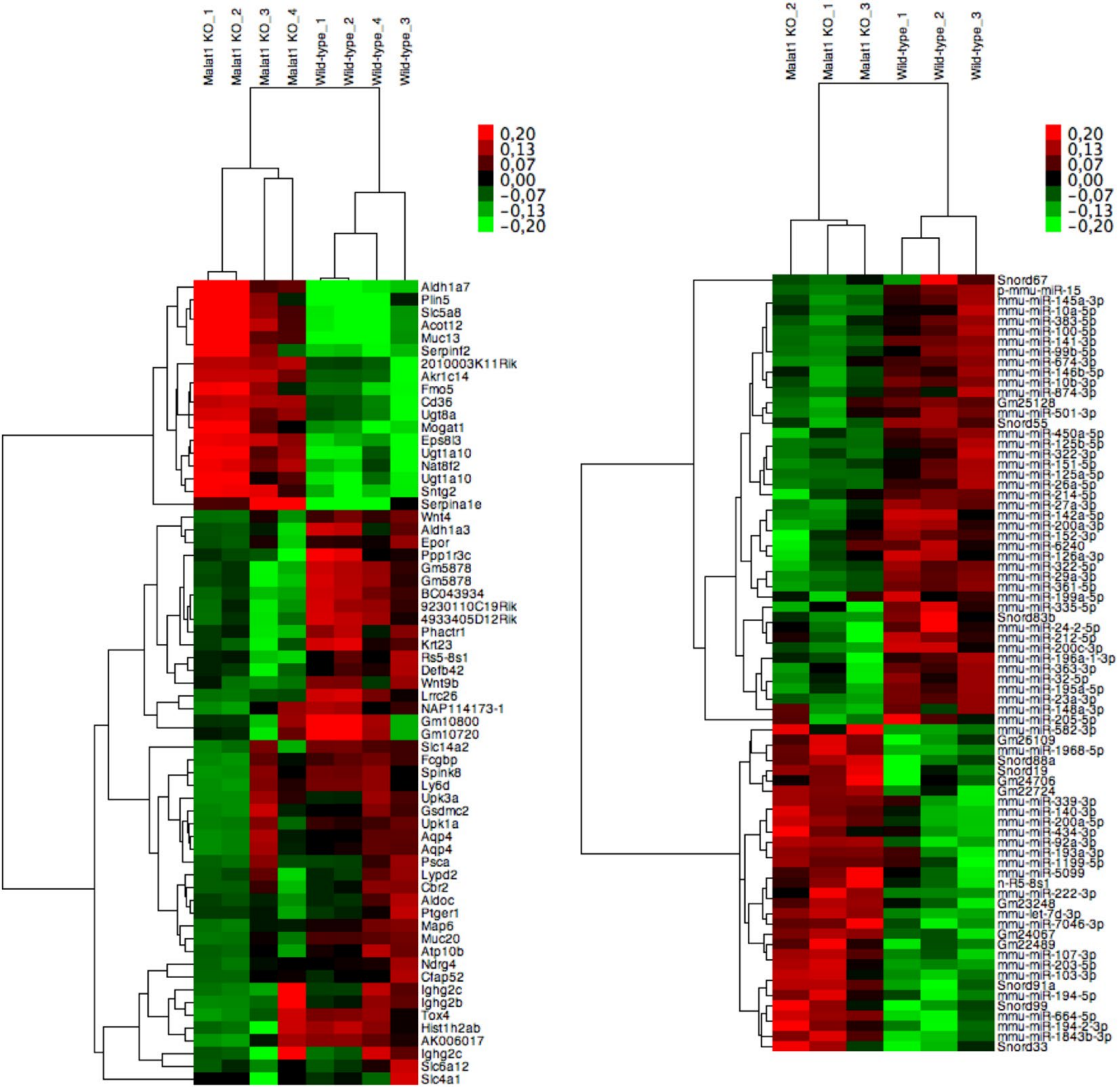
Heatmap of hierarchically clustered mRNA-array data in ischemic kidneys of *Malat1* knockout and wildtype mice (**A**): Signal intensities were log_2_ transformed, centered and normalized. Data was clustered using Spearman rank correlation and average linkage. Heatmap of hierarchically clustered small RNA-sequencing data in ischemic kidneys of *Malat1* knockout and wildtype mice (**B**): Signal intensities were log_2_ transformed, centered and normalized. Data was clustered using Spearman rank correlation and average linkage.

In order to find potentially dysregulated signalling pathways and small RNA expression levels *in vivo* we performed a whole genome mRNA array as well as a small RNA sequencing approach in ischemic kidneys of *Malat1* knockout and wildytpe mice. The data was subsequently filtered for differentially expressed mRNA and small RNA. Passed candidates were finally hierarchically clustered and displayed as heatmap (Fig. 8A for mRNA and B for small RNA). Additionally, most highly regulated candidates are summarized in Supplemental Table 1 (mRNA) and Supplemental Table 2 (small RNA). Only minor differences in mRNA and small RNA expression levels were detected. *Epidermal* *growth* *factor* *receptor* *kinasesubstrate* *8-like* *protein* *3* (Eps8l3; log2 fold change of 1.4), *2010003K11Rik* (log2 fold change of 1.4) and *aldehyde* *dehydrogenase* *family* *1*, *subfamily* *A7* (Aldh1a7; log2 fold change of 1.2) were found to be the top upregulated candidates. *Gm5878* (log2 fold change of 3.9), *aldehyde* *dehydrogenase* *1* *familymember* *A3* (Aldh1a3; log2 fold change of FC −3.0) and solute carrier family 14 (urea transporter), member 2 (Slc14a2 log2 fold change of 2.7) were top downregulated candidates. These candidates were validated by qPCR (see Supplemental Fig. 4A–F). Small RNA sequencing revealed several transcripts to be slightly regulated: miR-15 (log2 fold change 1.5), miR-383–5p (log2 fold change of 1.3) and miR-146b-5p (log2 fold change of 1.1) were top upregulated candidates, while Gm24706 (log2 fold change of 1.4), miR-7046–3p (log2 fold change of 1.1) and miR-203–5p (log2 fold change of 0.9) were top downregulated transcripts. Supplemental Fig. 4G shows PANTHER gene ontology enrichment analysis of dysregulated gene expression. Gene ontology analysis revealed the 5-Hydroxytryptamin degradation as the strongest influenced pathway, biological adhesion processes as the mainly affected biological processes, antioxidant activity as the primarily controlled molecular function and cell adhesion molecules as the mostly affected protein class.

## Discussion

In the present study we investigated the *in vivo* role of the lncRNA *Malat1* on the induction and progression of renal I/R-injury. While we did detect increased expression levels of *Malat1* in ischemic kidney biopsies and plasma of patients with AKI, ischemic mouse kidneys and sorted endothelial cells and tubular epithelial cells *ex vivo*, we were unable to report an overt phenotype of *Malat1* knockout mice in contrast to previous studies investigating the *in vivo* role of *Malat1* in other disease settings. More specifically, we found that in unilateral renal I/R-injury *Malat1* knockout mice exhibited the same degree of outer medullary injury, capillary rarefaction, fibrosis, infiltration of inflammatory cells, inflammatory gene expression and proliferation as did *Malat1* wildtype mice. In bilateral renal I/R-injury *Malat1* knockout mice showed a similar level of kidney dysfunction and survival as *Malat1* wildtype mice.

The role of microRNAs in the kidney has been extensively studied over the past 10 years. Very limited information is available on the role and functional importance of lncRNAs. We here provide the first study investigating the role of a lncRNA in experimental acute kidney injury. It is becoming evident that lncRNAs are important epigenetic regulators of tissue homeostasis during development and disease^4^. Although the function of most lncRNAs is unexplored to date, a mechanistic annotation has been suggested by Howard Chang’s group^4,12^. Here, lncRNAs were described to either function as (1) *signal lncRNAs*, which are spatiotemporally transcribed in response to developmental cues or cellular context; (2) *decoy lncRNAs*, which impact on transcriptional regulation by titrating away transcription factors and other proteins from chromatin; (3) *guide lncRNAs*, which sequester ribonucleoprotein complexes and direct them to chromatin; (4) *scaffold lncRNAs*, which interact with multiple partners to form a chromatin modifying complex.

A few lncRNAs have thus far been functionally characterized. For instance, the long intergenic RNA (lincRNA) *Air* and X-chromosome inactivation by *Xist* (X-inactive specific transcript) play a critical role in the regulation of imprinting^13^. The lncRNA *H19* is imprinted with maternal expression^14^. It has been shown to have a tumour-suppressive effect both *in vitro* and *in vivo*^15^. A lncRNA termed cardiac hypertrophy associated transcript (Chast) was found to be increased in mice and humans with cardiac hypertrophy^16^. Gain- and loss-of function studies underlined the importance of this transcript for cardiac hypertrophy and fibrosis development. RNA-sequencing analysis in a UUO mouse model of kidney fibrosis recently revealed a number of lncRNAs to be highly dysregulated^17^.

*Malat1* was first described in metastasizing non-small cell lung cancer, hence its name^18^. Owing to the high degree of conservation between different species and high expression levels *Malat1* knockout mice have been generated to further elucidate the role of this lncRNA. Abnormalities during embryonic or post-natal development have not been detected^19–21^, indicating that *Malat1* may only have a major role under pathological conditions. *Malat1* has been demonstrated to sequester serine/arginine (SR) splicing factors in nuclear speckle domains, thereby regulating alternative splicing^4,5^. In endothelial cells hypoxic stress has been shown to induce the expression of *Malat1*, leading to enhanced proliferation and migration as well as a reduction of apoptosis^8^. *In vivo*, *Malat1* knockout mice were characterized by impaired proliferation of endothelial cells and reduced vascularization in the developing retina^8^. In a hindlimb ischemia model, pharmacological silencing of *Malat1* reduced blood flow recovery during reperfusion and capillary density^8^. High glucose treatment induced the expression of *Malat1* in endothelial cells. This was paralleled by induction of inflammatory gene expression^6^. In another study *Malat1* was found to be increased in the cortex of mice with diabetic nephropathy as well as in cultured podocytes under high glucose conditions^22^. *Malat1* silencing restored podocyte function *in vitro*^22^. In diabetic rats *Malat1* was highly upregulated in the kidney. Here, *Malat1* influenced pyroptosis by sponging miR-23c^23^. Recently, the induction of *Malat1* by inspiratory hypoxia was assessed in mice^9^. It was shown that the induction was most pronounced in kidney and testis. Hypoxic induction in the kidney was confined to proximal rather than distal tubular epithelial cells as shown by *in situ* hybridization^9^. Direct oxygen-dependent regulation of *Malat1* was further validated in isolated primary kidney epithelial cells^9^. However, most of the available literature did not assess the *in vivo* role of *Malat1* by using *Malat1* knockout mice, which are available^19–21^.

In contrast to the aforementioned studies the absence of *Malat1* does not seem to impact on the resolution of disease. In a study involving genotoxic stress using diethylnitrosamine (DEN) to induce liver cancer *Malat1* knockout mice and littermate controls were investigated^24^. Here, *Malat1* knockout mice did not differ with respect to tumor development after 1 year compared to controls. In thoracic aortic constriction (TAC), a model of cardiac pressure overload, *Malat1* knockout mice were investigated compared to wildtype controls^25^. Absence of *Malat1* did not affect cardiac hypertrophy upon pressure overload. There are numerous *in vitro* studies claiming a role for *Malat1* in liver cancer progression^26–28^. In addition, in blood samples of patients with myocardial infarction *Malat1* was shown to be regulated^29^. *Malat1* was shown to impact on proliferation of cardiomyocytes *in vitro*^30^. However, in the *in vivo* studies referenced above changes between wildtype and *Malat1* knockout animals were not detected^24,25^. Taking into account these *in vitro* and biomarkers studies as well as our results of increased expression levels in hypoxic cells and blood of patients, we believe, that *Malat1* may have a role as a biomarker of different pathologies, including AKI. Most importantly, however, its role *in vivo* is either minimal or masked by redundant and/or overwhelming mechanisms. Whole genome expression analysis as well as small RNA sequencing revealed only minimal differences between the two groups. Most highly dysregulated genes included *Epidermal growth factor receptor kinase substrate 8-like protein 3*, *2010003K11Rik* and *aldehyde dehydrogenase family 1*, *subfamily A7*. Intriguingly, *ALDHs* are described to be upregulated in response to oxidative stress to prevent further impairment and are associated with disease, in which oxidative stress plays an important role, e.g. diabetes and acute lung injury^31^. Most highly downregulated candidates were *Gm5878*, *aldehyde dehydrogenase 1 family member A3* and solute carrier family 14 (urea transporter), member 2. Small RNA sequencing revealed miR-15, miR-383-5p and miR-146b-5p as top upregulated candidates. Interestingly, miR-15 is described to play a role in apoptosis by targeting Bcl2 in chronic lymphocytic leukemia (CLL)^32^ and mir-146b-5p was found to be induced in AKI and fibrosis^33^. Top downregulated small RNA candidates in our sequencing analysis were Gm24706, miR-7046-3p and miR-203-5p. Although we identified several mRNA and small RNA with potential impact on oxidative cellular stress and apoptosis induction to be slightly dysregulated and to be in association with AKI and fibrosis, the differences were only marginal and indicate *Malat1* loss to only have a minor influence *in vivo*.

It has previously been shown, that *Malat1* affects hind limb ischemia^8^. In contrast to this study, in which *Malat1* was pharmacologically silenced by GapmeRs we used mice with a genetic deletion of *Malat1*. Compensatory mechanisms might be activated in *Malat1* knockout mice during the embryonic developmental phase, which might cause possible adaptive response mechanisms, which could contribute to the absence of discernable effects in our study. Inducible knockout strategies could be used to bypass this effect and to identify possible compensatory mechanisms.

In conclusion, using two models of renal I/R-injury we did not detect a disease-specific phenotype of *Malat1* knockout mice. *Malat1* may be a viable biomarker of I/R-injury of the kidney, as evidenced by increased levels in plasma and kidney biopsies of patients in our study. However, its role *in vivo* on relevant signalling pathways is likely limited.

## Concise Methods

### Patients with AKI

Plasma samples of patients with AKI were collected as part of the HANDOUT trial^34^. In brief, in this study patients in seven ICUs of the tertiary care centre at the Hannover Medical School suffering from AKI were evaluated for inclusion. The study protocol was approved by the Hannover Medical School Ethics Committee and was conducted in accordance with the declaration of Helsinki and German Federal Guidelines. Written informed consent was obtained by the patient or his/her legal representative. The inclusion criteria were non-post-renal AKI with RRT dependence indicated by the loss of kidney function of >30% calculated estimated glomerular filtration rate (eGFR) with either the Modification of Diet in Renal Disease (MDRD) or Cockcroft–Gault equation and/or cystatin C-GFR within 48 hours prior to inclusion and oliguria/anuria (<30 mL/h >6 hours prior to inclusion) or hyperkalemia (>6.5 mmol/L) or severe metabolic acidosis (pH <7.15, bicarbonate <12). Exclusion criteria were pre-existing chronic kidney disease as defined by eGFR <60 mL/min or a serum creatinine concentration >1.7 mg/dL more than 10 days prior to initiation of the first RRT. Patient characteristics are shown in Table 1.

### Kidney biopsies of patients

In kidney transplant recipients, who presented with prolonged (707 ± 73 minutes, n = 5) and short (208 ± 56 minutes, n = 5) cold ischemia time (CIT), renal biopsy specimens and clinical and demographic data were collected. Patient characteristics are shown in Table 2. The study protocol was approved by the Hannover Medical School Ethics Committee (“Ethikkommission der Medizinischen Hochschule Hannover”) and was conducted in accordance with the declaration of Helsinki and German Federal Guidelines. Written informed consent was obtained by the patient or his/her legal representative.

### Animals

Male *Malat1* knockout mice and littermate wildtype control mice (both on a C57BL/6 background) were housed under standard conditions^20^. These mice were previously generated and described^20^. These mice are viable and fertile and do not display a pathological phenotype under basal conditions. Mice that were 10 to 12 weeks old weighing between 20 and 30 g were used for all experiments. In unilateral I/R-injury experiments n = 6 animals per group were included, in bilateral I/R-injury experiments 10 animals per group were included. All animal experimental procedures were in agreement with institutional and legislational regulations and were approved by local authorities of lower Saxony (“Niedersächsisches Landesamt für Verbraucherschutz und Lebensmittelsicherheit”; approval number 13/1245).

### Genotyping of mice

As described previously^20^ a three-primer-PCR strategy was used for genotyping of *Malat1* knockout mice. Primer 1 (CACTCTGGGAATGTTTTTGG), Primer 2 (CAGGAAAACGCAAAAGGTGT) and Primer 3 (TGTCGAAAAGAGGTGGTGTG) produced a 120 bp fragment for the wild-type allele and a 204 bp fragment for the *Malat1*-deleted locus. PCR conditions were as follows: 4 min 95 °C, followed by 30 cycles of 30 sec. 95 °C, 30 sec. 56 °C, 30 sec. 72 °C, and a final elongation for 10 min at 72 °C. Genotyping analyses of *Malat1* wildtype and knockout mice are shown in Supplemental Fig. 4E. *Malat1* absence was also confirmed by qPCR (Supplemental Fig. 1F).

## Electronic supplementary material

Supplemental Materials and Methods
