# Supplementary material for: Hypoxia-induced long non-coding RNA Malat1 is dispensable for renal ischemia/reperfusion-injury
Source: Sci Rep. 2018 Feb 21;8:3438. doi: 10.1038/s41598-018-21720-3 (PMC5821887; doi:10.1038/s41598-018-21720-3)

Hypoxia-induced long non-coding RNA *Malat1* is dispensable for renal ischemia/reperfusion-injury

Malte Koelling[1], Celina Genschel[2], Tamas Kaucsar[5], Anika Huebner[2], Song Rong[3], Roland Schmitt[3], Inga Soerensen-Zender[3], George Haddad[1], Andreas Kistler[9], Harald Seeger[1], Jan T. Kielstein[7], Danilo Fliser[8], Hermann Haller[3], Rudolf Wuethrich[1], Martin Zoernig[6], Thomas Thum[2,4,10] and Johan Lorenzen[1]

[1]Department of Nephrology, University Hospital Zürich, Switzerland, [2]Institute of Molecular and Translational Therapeutic Strategies (IMTTS), Hannover Medical School, Germany, [3]Department of Nephrology, Hannover Medical School, Germany, [4]National Heart and Lung Institute, Imperial College London, U.K., [5]Semmelweis University, Budapest, Hungary, [6]Georg-Speyer-Haus, Institute for Tumor Biology and Experimental Therapy, Frankfurt, Germany, [7]Department of Nephrology, Städtisches Klinikum Braunschweig GmbH, Braunschweig, Germany, [8]Saarland University Medical Centre, Homburg/Saar, Germany, [9]Department of Internal Medicine, Cantonal Hospital Frauenfeld, Frauenfeld, Switzerland, [10]Excellence Cluster REBIRTH, Hannover Medical School, Hannover, Germany

Supplemental Materials and Methods

Ischemic/Reperfusion injury Protocol

Clamping of renal pedicles was applied to induce significant renal I/R injury as described previously [1]. Following isoflurane anaesthesia male C57BL/6 mice were subjected to median laparotomy, thereafter renal pedicles were dissected and a vascular clamp was applied for 30 minutes.

For the survival analysis sham operated animals (n = 4) were also included. Blood samples for analysis of renal function parameters (serum-urea and –creatinine) were drawn on days 0, 1, 3 and 7 (analysed on a Beckman Analyzer, Beckman Instruments GmbH, Munich, Germany). In a second group of mice the renal pedicle was only clamped on the left side (unilateral I/R-injury). In this setting the contralateral kidney serves as an internal control to the injured kidney (I/R-kidney). These animals were sacrificed on day 1 and day 7 after renal IR injury and kidneys were harvested for further examination. At each time point 6 mice were used for unilateral I/R-injury. In bilateral I/R-injury studies 10 mice were used per group. *In vivo* studies conformed to the *Guide for the Care and Use of Laboratory Animals* published by the US National Institutes of Health (NIH publication No. 85-23, revised 1996).

Histology, Immunostaining

After kidney extraction, a representative part of each kidney was fixed immediately in PBS-buffered 4% paraformaldehyde and embedded in paraffin. Certain immunostainings were also performed in cryosections. Four-micrometer sections were used for immunostaining and for hematoxylin/eosin staining to evaluate histologic damage.

The severity of morphologic renal damage was assessed in a blinded manner using an arbitrary score based on PAS-stained kidney sections following a modification of a protocol developed by Broekema et al [2]. Briefly, the extent of four typical I/R injury–associated damage markers (i.e., dilatation, denudation, intraluminal casts, loss of brush border membrane and cell flattening) was expressed in arbitrary units (AU) in a range of 0 to 4 according to the percentage of damaged tubules: 0, no damage; 1, less than 25% damage; 2, 25%–50% damage; 3, 50%–75% damage; and 4, more than 75% damage. Immunostainings for inflammatory cell influx was performed using the following primary antibodies: monoclonal rat anti-mouse F4/80 (Serotec, Oxford, United Kingdom), monoclonal rabbit anti-Mouse CD3 (Abcam, Cambridge, UK). Analyses of capillary rarefaction in outer medulla were evaluated after fluorescent immunohistochemical staining for polyclonal rabbit anti-mouse CD31 (Abcam,

Cambridge, UK). Immunostainings for detection of proliferating cells was performed using the following primary antibody: polyclonal rabbit anti-mouse Ki67 (Abcam, Cambridge, UK). Deparaffinized kidney sections were boiled in citrate buffer for antigen retrieval, blocked with 5% milk, and incubated overnight at 4°C with primary antibodies. This was followed by antibody visualization using Alexa 594 secondary antibodies (Molecular Probes/Invitrogen, Carlsbad, CA). Quantification of F4/80-, CD3-, CD31- and Ki67 expressing cells was done by counting of positive cells in five randomly chosen, non-overlapping fields in outer medulla.

Cell Culture

For *in vitro* analyses immortalized human proximal tubular cells (HK-2 cells) and human umbilical vein endothelial cells (HUVECs) were used. HUVECs were cultured in endothelial basal medium-2 (EBM2) culture medium supplemented with EGM SingleQuots (Cambrex, Verviers, Belgium), 10% FCS and 1% Penicillin/Streptomycin. HK-2 cells were cultured in DMEM medium supplemented with 10% FCS and 1% Penicillin/Streptomycin. Cells were grown to 60% to 70% confluence and used for further downstream analyses. Cells were subjected to hypoxia (0.1% O_2) for 24 hours and reoxygenation for 2 hours. Apoptosis was determined by Caspase 3/7 Glo assay according to the manufactures instruction. Briefly, this assay is luminescent based measuring the caspase-3 and -7 activities in cultured adherent cells. The proluminescent caspase-3/7 substrate consists of the tetrapeptide sequence DEVD. If this substrate is cleaved, aminoluciferin is released to produce light. In addition, Annexin Apoptosis Assay was performed according to the manufactures instruction (FlowCellectTM Annexin Red Kit, Millipore). Briefly, cells undergoing early apoptosis were detected with Annexin V+ and 7-amino actinomycin D (7-AAD)- stainings using fluorescence-activated cell sorting (FACS) on a Guava easyCyteTM sorter (Millipore) using Cytosoft software (Guava Technologies) according to the manufacturer’s instructions.

Protein Analysis

Western blot analysis was performed using 10 to 40 µg of total protein. Cell lysis was performed (cell lysis buffer, Cell Signaling Technology) and protein electrophoresis was initiated. Proteins were transferred to polyvinylidene difluoride (PVDF) membranes, blocked with 5% milk in TBS-Tween, and probed overnight at 4°C with the following primary antibodies: Anti-HIF-1α antibody (Abcam) and Anti-β-Actin (Sigma-Aldrich) was used as an internal loading control and for normalization of protein

quantification. Immunoblots were scanned and quantified using ImageJ densitometry software.

Subcellular fractionation of cells

To determine subcellular localization of *Malat1* we employed a protocol described previously by Cabianca and co-workers[3]. Here, the cytoplasmic fraction was separated from the nuclear-soluble and nuclear chromatin associated fraction. Briefly, cells trypsinized and centrifuged. The pellet was lysed with 175 µl of cold RLN1 solution (50 mM Tris HCl pH 8.0; 140 mM NaCl; 1.5 mM MgCl2; 0,5% NP-40; 2mM Vanadyl Ribonucleoside Complex; Sigma) and incubated for 5 min on ice. Next, the suspension was centrifuged at 4° C and 300 g for 2 min and the supernatant, corresponding to the cytoplasmic fraction, was transferred into a new tube and stored on ice. The pellet containing nuclei was extracted with 175 µl of cold RLN2 solution (50 mM Tris HCl pH 8.0; 500 mM NaCl; 1.5 mM MgCl2; 0,5% NP-40; 2mM Vanadyl Ribonucleoside Complex) and incubated for 5 min on ice. The suspension was centrifuged at 4° C and 16360 g for 2 min and the supernatant, corresponding to the nuclear-soluble fraction, was transferred into a new tube and stored on ice. The remaining pellet corresponds to the chromatin-associated fraction. Cytosolic expression might suggest a function as competing endogenous RNA regarding sponging of microRNAs, while nuclear expression suggests interaction with chromatin modifying factors or transcription factors.

Chromatin immunoprecipitation (ChIP)

ChIP was performed using the MAGnify Chromatin Immunoprecipitation System (Thermo Fisher Scientific) according to the manufacturer' instructions. Cells (1×10^7) were cross-linked in 1% formaldehyde for 10 min. After cell lysis, the chromatin was sheared using a sonicator to an average size of 500 bp and enriched with an antibody against HIF-1α or with isotype IgG coupled to magnetic Dynabeads (Invitrogen) at 4°C overnight. Subsequently, crosslinks were reversed, the DNA cleaned by RNase A (0.2 mg/mL) and proteinase K (2 mg/mL) and purified by phenol/chloroform. The specific sequence from immunoprecipitated and input DNA were determined by PCR primers for *Malat1* promoter upstream region (see primer list in **Supplemental Table 3**).

Cell cycle analysis

To detect cells in the different phases of the cell cycle, cellular DNA was labeled with propidium iodide using Guava Cell Cycle Reagent (Guava Technologies). Labeled

cells were analysed using fluorescence-activated cell sorting (FACS) on a Guava easyCyte sorter (Millipore) using the Cytosoft software (Guava Technologies) according to the manufacturer's instructions.

Proliferation Assay

To measure the proliferation rate *in vitro,* Bromodeoxyuridine (BrdU) Cell Proliferation ELISA Kit (Abcam) was used according to the manufacturer's instructions.

Ex vivo cell purification

The cellular origin of *Malat1* following induction of I/R-injury was investigated by fluorescence-associated cell sorting (FACS) analysis using specific antibodies following a protocol by Chau et al. with modifications [1,4]. Following clamping of the right renal pedicle and a reperfusion period of 24 hours both kidneys were extracted, de-capsulated, homogenized, then incubated at 37ºC for 30 min with Collagenase II (81 U/ml) and DNase (100U/ml, Roche) in serum free DMEM. After centrifugation, cells were re-suspended in 5ml of PBS/1% BSA, and filtered (40µm). Cells were separated using the following specific antibodies or lectins: rat anti-mouse-CD31-PE (1:400 BD Pharmingen) for endothelial cells, Lotus teragonolobus lectin (1:200, DAKO) for proximal epithelium and anti-mouse Kim-1-biotin (1:200, E Bioscience) followed by streptavidin-APC (1:2000, BD Pharmingen) for injured proximal epithelium. Subsequently, RNA was isolated by Trizol method.

Scratch wound healing assay

Transfected HUVECs were cultivated in Endothelial Basal Medium with supplements at 37°C, 5% CO2. The scratches in the cell monolayer were generated with a 100-µl tip, and the cells were photographed at 0, 8, and 24 hours with a Nikon Ti 90 microscope (Germany). Subsequently, the cell free area was calculated.

Reverse Transcription–Polymerase Chain Reaction, and Global Transcriptome Analysis

RNA isolation was performed with TRIzol reagent (Invitrogen) according to the manufacturer's instructions. Reverse transcription–polymerase chain reaction analysis was performed in an ICycler (Bio-Rad). Gene array analysis was performed with the Affymetrix GeneChip system according to the manufacturer's instructions (Affymetrix Systems). Next Generation Small RNA Sequencing on an Ilumina platform was performed at Helmholtz Zentrum for Infection Biology, Braunschweig, Germany. A total of 24,410 protein-coding genes and 564 small RNAs were detected in our samples.

Reverse transcription was performed with total RNA using oligoDT primers (Bio-Rad). Amplified cDNA was used as a template for quantitative PCR. Reverse transcription polymerase chain reaction analysis was performed in an ICycler (Bio-Rad) with SYBRgreen mastermix. The specific cel-miR-39 primer was purchased from Thermo Fisher Scientific. A list of primers used can be found in **Supplemental Table 3**.

Transfection Assays

Transient liposomal transfection of antisense oligonucleotides targeting *Malat1* (GapmeRs) was performed according to the manufacturers' instructions (Exiqon, Denmark). Briefly, cells were split 1 day before transfection to reach 60% to 70% confluence on the day of transfection. Specific GapmeRs targeting *Malat1* as well as control GapmeRs and Lipofectamine 2000 (Invitrogen) were mixed separately and incubated for 5 minutes with Opti-MEM I media (Invitrogen). Complexes were added together and incubated for 20 minutes. Media were changed to antibiotic-free media before the addition of liposomal antisense complexes (final concentration 150 nmol/L). Cells were incubated for 4 hours before the media were changed to fresh media. Specific assays were performed after 48 hours. **Supplemental Figure 1G** shows *Malat1* GapmeR knockdown efficiency.

Supplemental Figure Legends

Supplemental Figure 1: Hif-1α immunoblotting in normoxia, 24h of hypoxia, 48h of hypoxia and 72h of hypoxia and quantification of the results **(A)**. Gene expression analysis of Hif-1α target genes CA9 **(B)**, GLUT1 **(C)** and PHD2 **(D)** after 24h of hypoxia. *P<0.05; **P<0.01; ***P<0.001; ****P<0.0001. Genotyping of homozygous wildtype **(Hz WT)**, heterozygous **(het)** and homozygous Malat1 knockout **(Hz KO)** mice **(E).** *Malat1* gene expression in mouse kidneys of homozygous wildtype and homozygous *Malat1* knockout mice **(F)**. *Malat1* GapmeR knockdown efficiency **(G)**.

Supplemental Figure 2: *Malat1* dependent apoptosis induction in HUVEC upon hypoxia. Annexin/7AAD staining in GapmeR Control and GapmeR Malat1 treated HUVEC after hypoxia/reoxygenation (A) (n=5). Caspase 3/7 acticity in GapmeR Control and GapmeR Malat1 treated HUVEC after hypoxia/reoxygenation (B) (n=5). *P<0.05; **P<0.01; ***P<0.001; ****P<0.0001.

Supplemental Figure 3: Functional role of *Malat1* in HK-2 cell biology. HK-2 cells detected in G0/G1 phase (A), S phase (B), and G2/M phase (C) under basal conditions (n = 5). HK-2 cells detected in G0/G1 phase (D), S phase (E), and G2/M phase (F) after hypoxia/reoxygenation (n = 5). Annexin/7AAD staining in GapmeR Control and GapmeR *Malat1* treated HK-2 after hypoxia/reoxygenation (I) (n=5). Caspase 3/7 acticity in GapmeR Control and GapmeR Malat1 treated HK-2 after hypoxia/reoxygenation (J) (n=5). *P<0.05; **P<0.01; ***P<0.001; ****P<0.0001.

Supplemental Figure 4: Whole genome mRNA validation by quantitative realtime PCR: **(A)** Epidermal growth factor receptor kinase substrate 8-like protein 3 (Eps8l3), **(B)** 2010003K11Rik, **(C)** aldehyde dehydrogenase family 1, subfamily A7 (Aldh1a7), **(D)** Gm5878, **(E)** aldehyde dehydrogenase 1 family member A3 (Aldh1a3) and **(F)** solute carrier family 14 (urea transporter), member 2 (Slc14a2). PANTHER gene ontology enrichment analysis of deregulated genes in the whole genome expression array **(G)**: Signaling pathway analysis, biological processes, molecular functions and protein classes. *P<0.05; **P<0.01; ***P<0.001; ****P<0.0001.

Supplemental Table 1: mRNA Microarray Data

mRNA	FC	p-value	KO 1	KO 2	KO 3	KO 4	WT 1	WT 2	WT 3	WT 4
Eps8l3	1.4	**	11.5	11.4	11.3	10.5	10.1	10.2	8.8	10.1
2010003K11Rik	1.3	*	11.9	11.9	11.9	11.2	11.2	11.2	8.6	10.9
Aldh1a7	1.2	**	12.9	12.9	12.0	11.6	11.3	11.3	10.7	11.1
Akr1c14	1.2	*	9.0	9.0	9.1	8.1	8.2	8.2	6.1	7.9
Ugt1a10	1.2	*	10.7	10.8	9.6	9.4	9.2	9.1	8.2	9.3
Slc5a8	1.2	**	10.5	10.3	9.8	9.2	9.0	8.9	8.7	8.4
Muc13	1.2	**	9.2	9.2	8.4	8.0	7.7	7.7	7.5	7.3
Fmo5	1.1	*	12.5	12.6	12.0	10.9	11.3	11.4	9.9	10.8
Plin5	1.1	*	10.3	10.2	9.7	8.7	8.6	8.6	9.2	8.0
Ugt8a	1.1	*	10.9	11.0	10.4	10.1	10.1	10.1	8.0	9.7
Cd36	1.1	*	12.9	13.0	12.9	12.3	12.3	12.2	10.3	11.9
Acot12	1.1	**	12.7	12.7	12.4	11.7	11.5	11.5	11.2	10.9
Mogat1	1.1	*	13.8	13.9	13.0	12.4	12.6	12.6	11.6	12.0
Serpina1e	1.1	*	11.3	11.3	12.7	12.3	10.8	10.8	11.8	9.9
Nat8f2	1.1	*	9.0	8.9	8.5	8.2	7.9	7.8	6.6	8.1
Serpinf2	1.1	*	15.3	15.4	14.7	13.7	14.0	14.0	13.3	13.6
Ugt1a10	1.0	**	11.9	11.9	11.3	11.1	10.7	10.7	9.8	10.9
Sntg2	1.0	**	8.3	8.3	8.2	7.4	7.3	7.1	6.5	7.1
Gm5878	-3.9	****	11.2	11.5	11.4	11.4	15.4	15.3	15.1	15.3
Aldh1a3	-3.0	***	6.8	6.7	8.4	6.8	10.6	10.5	11.0	8.7
Gm5878	-2.8	****	7.6	7.7	7.7	7.6	10.6	10.5	10.2	10.5
Slc14a2	-2.7	**	6.5	6.6	9.7	7.1	10.1	10.1	10.6	9.9
Ighg2c	-1.9	*	8.1	8.0	8.3	10.9	9.6	9.8	11.8	11.9
BC043934	-1.9	****	12.6	13.1	12.9	14.8	14.8	14.8	14.8	14.6
Ighg2c	-1.8	*	8.1	8.0	9.0	10.8	9.9	10.0	11.6	11.6
Fcgbp	-1.7	**	6.9	6.8	9.0	7.9	9.1	9.3	9.7	9.5
Lrrc26	-1.6	****	6.2	6.2	7.1	6.8	8.3	8.4	7.9	8.1
Lypd2	-1.6	*	7.2	7.4	9.0	7.3	8.7	8.6	10.7	9.3
Ighg2b	-1.6	*	7.1	7.0	8.2	9.3	8.8	8.9	10.2	9.8
Spink8	-1.4	**	10.6	10.6	12.7	11.6	12.8	12.8	12.6	13.1
Tox4	-1.4	**	8.7	8.8	9.8	10.3	10.8	10.9	10.6	11.1
Wnt4	-1.4	**	7.8	7.8	9.0	8.1	9.3	9.5	10.2	9.3
Upk3a	-1.4	*	6.7	6.6	9.0	7.9	8.4	8.4	9.5	9.4

Ndrg4	-1.3	**	7.8	7.7	8.8	8.4	9.1	9.2	10.6	9.2
Upk1a	-1.3	**	7.8	7.8	9.5	8.3	9.5	9.4	10.3	9.5
Ppp1r3c	-1.3	***	6.4	6.2	6.8	6.1	8.1	7.9	7.5	7.2
Gm10800	-1.3	*	7.5	7.5	7.7	8.5	9.7	9.7	7.3	9.5
Rs5-8s1	-1.2	**	13.7	13.7	13.7	13.4	14.4	14.7	15.8	14.4
Ly6d	-1.2	*	8.2	8.1	10.0	9.2	10.1	10.2	9.8	10.4
NAP114173-1	-1.2	**	7.6	7.4	8.7	8.7	9.5	9.5	9.5	8.9
Aqp4	-1.2	*	8.0	8.1	10.1	8.9	9.6	9.6	10.5	10.0
Aldoc	-1.2	**	10.3	10.2	10.9	10.3	11.2	11.1	12.7	11.5
Cfap52	-1.2	*	6.1	6.1	7.1	6.8	7.2	7.4	9.0	7.3
Gsdmc2	-1.2	*	8.5	8.4	10.6	9.2	10.0	10.1	10.7	10.6
Krt23	-1.2	****	9.3	9.1	9.3	9.5	10.6	10.6	10.6	10.1
9230110C19Rik	-1.2	****	14.7	15.0	15.0	15.0	16.2	16.1	16.1	16.1
Cbr2	-1.2	**	8.7	8.7	9.8	8.8	9.7	9.8	10.9	10.3
Defb42	-1.1	**	8.3	8.2	8.2	8.3	9.0	9.2	10.2	9.1
Psca	-1.1	*	6.1	6.0	7.7	6.6	7.2	7.2	8.9	7.6
Epor	-1.1	**	8.1	8.0	9.0	8.1	9.2	9.2	10.2	9.1
Wnt9b	-1.1	**	6.8	6.4	6.9	6.7	7.8	7.8	8.5	7.2
Muc20	-1.1	**	10.5	10.5	11.5	10.9	11.8	11.8	12.4	11.9
Ptger1	-1.1	*	7.3	7.3	8.0	7.3	8.1	8.2	9.8	8.3
Gm10720	-1.1	*	8.2	8.1	8.1	8.7	10.0	10.0	7.9	9.7
Slc4a1	-1.1	*	8.4	8.4	8.0	8.5	9.0	9.1	10.9	8.7
Map6	-1.1	**	7.9	7.8	8.7	8.4	9.0	9.0	9.8	9.2
Phactr1	-1.1	***	8.5	8.6	8.5	8.4	9.5	9.6	10.0	9.1
Hist1h2ab	-1.1	***	11.2	11.1	11.3	12.1	12.5	12.6	12.3	12.5
AK006017	-1.1	**	6.5	6.5	7.5	7.6	8.1	8.1	8.0	8.1
Aqp4	-1.0	*	8.1	8.1	9.7	8.8	9.4	9.5	10.1	9.7

4933405D12Rik	-1.0	****	7.2	7.4	7.4	7.5	8.5	8.4	8.2	8.4
Slc6a12	-1.0	*	6.5	6.7	6.9	7.0	7.3	7.4	9.0	7.6
Atp10b	-1.0	**	7.6	7.5	8.3	7.7	8.5	8.5	9.2	9.0

Malat1 knock-out = KO; Wild-type = WT

**p<5x10^-2; **p<1x10^-2; ***p<1x10^-3; ****p<10^-4*

**Supplemental Table 2:** small RNA Sequencing Data								
**miR-**	**FC**	**p**	**KO 1**	**KO 2**	**KO 3**	**WT 1**	**WT 2**	**WT 3**
15	1.5	***	7.7	9.8	11.7	22.6	28.0	52.1
383-5p	1.3	**	12.8	20.6	29.1	39.0	49.2	88.0
146b-5p	1.1	***	343.3	642.8	667.2	1040.7	933.8	1897.8
100-5p	1.1	****	474.0	532.3	706.8	931.1	1051.4	1984.6
450a-5p	1.1	***	70.0	45.0	70.0	106.9	124.3	174.7
Snord67	1.1	*	6.8	8.8	12.8	9.1	39.5	23.9
141-3p	1.0	****	1842.1	2191.7	2588.2	4032.3	4099.6	5583.8
363-3p	1.0	*	15.4	9.8	10.5	23.6	21.2	36.9
212-5p	1.0	*	13.7	21.5	9.3	37.1	31.8	29.3
10b-3p	0.9	***	66.6	95.9	109.6	166.7	155.2	215.9
99b-5p	0.9	***	8044.1	9063.3	11631.3	13441.0	17502.0	23928.3
Snord83b	0.9	**	44.4	60.7	60.0	96.9	130.1	81.4
335-5p	0.9	**	62.3	38.2	42.0	84.2	107.9	81.4
145a-3p	0.8	***	441.5	582.2	624.0	825.1	893.4	1310.8
151-5p	0.8	***	2672.3	2555.7	3437.4	4115.6	4740.5	7047.6
Gm25128	0.8	*	17.9	24.5	40.8	48.0	51.1	56.4
125b-5p	0.8	***	2273.4	2265.1	2659.4	3482.5	3672.7	5724.9
10a-5p	0.8	**	333381.4	439288.8	420832.8	556411.0	551572.2	1043729.9
196a-1-3p	0.8	*	22.2	25.4	18.7	33.5	35.7	55.4
501-3p	0.7	**	193.0	227.0	328.9	356.0	442.3	481.8
322-3p	0.7	**	355.3	320.0	368.6	447.4	483.8	843.1
27a-3p	0.7	***	1810.6	2097.8	2591.7	3605.7	3445.3	3758.7
142a-5p	0.7	**	2327.2	2500.9	3331.2	4909.1	4961.2	3693.9
214-5p	0.7	*	35.9	25.4	46.7	54.3	58.8	67.3
322-5p	0.7	***	872.0	737.8	1022.9	1432.9	1284.6	1568.0
32-5p	0.7	**	98.2	81.2	81.7	138.6	116.6	170.4
26a-5p	0.7	**	86713.2	83079.8	96123.7	122887.7	122294.4	178516.9
200a-3p	0.6	**	2966.0	2840.4	4083.6	5400.9	5341.0	4810.2
205-5p	0.6	*	264.8	598.8	415.2	874.0	626.4	521.9
874-3p	0.6	*	132.4	145.8	172.6	207.4	178.3	312.5
Snord55	0.6	*	88.0	116.4	120.1	164.8	167.7	163.9
29a-3p	0.6	***	6114.0	6077.1	7294.6	9584.4	9108.1	10934.4
125a-5p	0.6	**	12959.9	13240.3	14747.9	17428.1	19463.2	25189.1
6240	0.6	*	872.8	643.8	1253.9	1330.5	1565.1	1266.3
148a-3p	0.6	*	2989.1	5439.2	3547.0	5843.8	4277.9	7763.8
126a-3p	0.5	*	5011.5	3916.7	5998.8	8022.1	7797.4	6145.9
24-2-5p	0.5	*	186.2	226.0	182.0	267.2	330.6	265.9
674-3p	0.5	*	80.3	85.1	112.1	122.3	126.3	155.2
199a-5p	0.5	*	424.5	645.8	762.8	1002.6	744.0	878.9
152-3p	0.5	*	438.1	327.8	513.2	607.8	567.6	593.5
195a-5p	0.4	*	643.1	566.5	612.4	800.7	756.5	928.8
200c-3p	0.4	*	284.4	315.1	307.9	425.7	429.8	372.2
361-5p	0.4	*	189.6	182.0	211.1	251.8	246.7	286.5
23a-3p	0.4	*	1079.5	1150.7	1157.1	1450.1	1409.0	1644.0
140-3p	-0.4	*	4259.9	4669.1	3944.8	3678.2	3117.6	2807.1
222-3p	-0.4	*	4109.6	2829.7	3284.6	2638.4	2568.3	2382.8
5099	-0.5	*	3025.8	2633.0	3330.1	2544.2	2221.4	1632.0
92a-3p	-0.5	**	35321.9	34747.5	32978.6	27155.6	23265.0	2323.2
Gm26109	-0.5	*	863.4	648.7	683.5	454.7	520.4	586.0
103-3p	-0.5	*	4483.7	4131.0	3241.4	2925.5	2657.0	2811.5

582-3p	-0.5	*	287.8	428.6	425.7	267.2	257.3	261.5
let-7d-3p	-0.5	*	1912.2	1663.4	1628.3	1274.4	1170.9	1109.0
Snord99	-0.6	*	1223.0	1393.3	930.8	718.2	794.1	882.2
339-3p	-0.6	*	215.2	220.1	196.0	189.3	132.0	98.7
Gm22489	-0.6	*	141.8	96.9	92.1	67.9	81.0	70.5
Snord19	-0.6	*	118.7	118.4	126.0	66.1	95.4	76.0
Snord88a	-0.6	*	93.1	77.3	87.5	46.2	58.8	62.9
434-3p	-0.6	*	131.5	169.3	100.3	112.3	81.0	64.0
194-5p	-0.6	**	12152.9	9182.7	8020.1	7283.9	5519.2	6149.2
n-R5-8s1	-0.6	**	1244.3	962.8	1351.9	907.5	790.3	537.1
Snord91a	-0.7	**	198.1	184.0	156.3	117.7	99.3	121.5
1968-5p	-0.7	*	59.8	43.1	43.2	29.9	28.9	29.3
664-5p	-0.7	*	55.5	57.7	47.8	30.8	28.0	35.8
1843b-3p	-0.7	**	161.4	122.3	110.8	75.2	75.2	84.6
Gm24067	-0.7	**	143.5	115.5	115.5	82.4	82.9	53.2
193a-3p	-0.7	*	582.5	574.4	515.6	495.4	290.1	173.6
Snord33	-0.8	*	82.0	91.0	49.0	36.2	38.6	52.1
200a-5p	-0.8	***	645.7	668.3	523.7	435.7	336.3	278.9
1199-5p	-0.8	*	95.7	108.6	84.0	82.4	53.0	17.4
107-3p	-0.8	***	410.8	348.3	292.8	211.9	225.5	149.7
Gm23248	-0.8	*	85.4	55.8	65.3	48.9	39.5	21.7
Gm22724	-0.9	**	64.9	65.6	60.7	34.4	43.4	20.6
194-2-3p	-0.9	*	49.5	64.6	37.3	29.0	21.2	27.1
203-5p	-0.9	***	227.2	180.0	127.1	95.0	96.4	79.2
7046-3p	-1.1	*	17.1	14.7	25.7	10.0	5.8	6.5
Gm24706	-1.4	***	101.6	48.9	166.8	15.0	54.0	31.5

Malat1 knock-out = KO; Wild-type = WT

*p<5x10^-2; **p<1x10^-2; ***p<1x10^-3; ****p<10^-4

**Supplemental Table 3.** Primer pairs used for gene expression analysis		
Gene Symbol	Gene name	Primer Pairs
Malat1	metastasis associated lung adenocarcinoma transcript 1 [*Mus musculus*]	Fwd: TAAGCGCTTGCCTCTGTCTT<br>Rev: CACCTGCATTCTGTGTGGTC
Malat1 promoter region	Metastasis associated lung adenocarcinoma transcript 1 [*Mus musculus*] HIF-1α promoter region	Fwd: GGCCTACTGGGCTGACATTA<br>Rev: GAAGAAATCCCTTCAGGATCA
ActB	beta-actin [*Mus musculus*]	Fwd: ATCAAGATCATTGCTCCTCCTG<br>Rev: AGGGTGTAAAACGCAGCTCA
Kim1	kidney injury molecule-1 [*Mus musculus*]	Fwd: AAACCAGAGATTCCCACA CG<br>Rev: GTCGTGGGTCTTCCTGTAGC
Il-1beta	interleukin 1 beta [*Mus musculus*]	Fwd: AGGTCCACGGGAAAGACACAGG<br>Rev: GGGCTGCTTCCAAACCTTTGAC
Cxcl2 (MIP2a)	chemokine (C-X-C motif) ligand 2 [*Mus musculus*]	Fwd: CCAAGGGTTGACTTCAAGAAC<br>Rev: AGCGAGGCACATCAGGTACG
Il-6	interleukin 6 [*Mus musculus*]	Fwd: GAGAAAAGAGTTGTGCAATG<br>Rev: ATTTTCAATAGGCAAATTTC
Ccl2 (MCP-1)	chemokine (C-C motif) ligand 2 (Monocyte chemoattractant protein-1) [*Mus musculus*]	Fwd: GGCTCAGCCAGATGCAGTTA<br>Rev: ACTACAGCTTCTTTGGGACA
Col1a2	collagen, type I, alpha 2 [*Mus musculus*]	Fwd: CAGAACATCACCTACCACTGCAA<br>Rev: TTCAACATCGTTGGAACCCTG
Col3a1	collagen, type III, alpha 1 [*Mus musculus*]	Fwd: TGACTGTCCCACGTAAGCAC<br>Rev: GAGGGCCATAGCTGAACTGA
Tgfb	transforming growth factor beta [*Mus musculus*]	Fwd: CAACAATTCCTGGCGTTACCTTGG<br>Rev: GAAAGCCCTGTATTCCGTCTCCTT
Eps8l3	EPS8-like 3 [*Mus musculus*]	Fwd: CTCGGGTCCCTAATGGGAAG<br>Rev: CTCTAATGAGTCATCCCCAGCA
2010003 K11Rik	RIKEN cDNA 2010003K11 gene [*Mus musculus*]	Fwd: ACAGTACCAGCAGCATGTGAAA<br>Rev: TGGTGTCCAACAAGGTCTCTG
Aldh1a7	aldehyde dehydrogenase family 1, subfamily A7 [*Mus musculus*]	Fwd: TTTGCTGAGCCTGTCACTTG<br>Rev: CTGCTCACCGAATCATGCCA
Gm5878	predicted gene 5878 [*Mus musculus*]	Fwd: CTGGACTTTTGGACACAGGC<br>Rev: AACACTGAAGATGGCCGAGA

Aldh1a3	aldehyde dehydrogenase family 1, subfamily A3 [*Mus musculus*]	Fwd: TATCAACAACGACTGGCACG<br>Rev: CCACATCGGGCTTATCTCCT
Slc14a2	solute carrier family 14 (urea transporter), member 2 [*Mus musculus*]	Fwd: AGGAGAGAAAGTGAGGTGTCC<br>Rev: AGGACCAGGGGCTTATCTTT
CA9	carbonic anhydrase 9 [*Homo sapiens*]	Fwd: GGGTGTCATCTGGACTGTGTT<br>Rev: CTTCTGTGCTGCCTTCTCATC
GLUT1	Glucose transporter 1 [*Homo sapiens*]	Fwd: TCACTGTGCTCCTGGTTCTG<br>Rev: CCTGTGCTCCTGAGAGATCC
PHD2	Prolyl Hydroxylase Domain-2 [*Homo sapiens*]	Fwd: GAAAGCCATGGTTGCTTGTT<br>Rev: TTGCCTTCTGGAAAAATTCG

Supplemental References

1. Lorenzen JM, Kaucsar T, Schauerte C, Schmitt R, Rong S, Hübner A, Scherf K, Fiedler J, Martino F, Kumarswamy R, Kölling M, Sörensen I, Hinz H, Heineke J, van Rooij E, Haller H, Thum T. MicroRNA-24 antagonism prevents renal ischemia reperfusion injury. *J Am Soc Nephrol*. 2014; 25(12):2717-29.
2. Broekema M, Harmsen MC, Koerts JA, Petersen AH, van Luyn MJ, Navis G, Popa ER. Determinants of tubular bone marrow-derived cell engraftment after renal ischemia/reperfusion in rats. *Kidney Int*. 2005; 68(6):2572-81.
3. Cabianca DS, Casa V, Bodega B, Xynos A, Ginelli E, Tanaka Y, Gabellini D. A long ncRNA links copy number variation to a polycomb/trithorax epigenetic switch in FSHD muscular dystrophy. Cell. 2012;149(4):819-31.
4. Chau BN, Xin C, Hartner J, Ren S, Castano AP, Linn G, Li J, Tran PT, Kaimal V, Huang X, Chang AN, Li S, Kalra A, Grafals M, Portilla D, MacKenna DA, Orkin SH, Duffield JS. MicroRNA-21 promotes fibrosis of the kidney by silencing metabolic pathways. *Sci Transl Med*. 2012; 4(121):121ra18.

Supplemental Figure 1

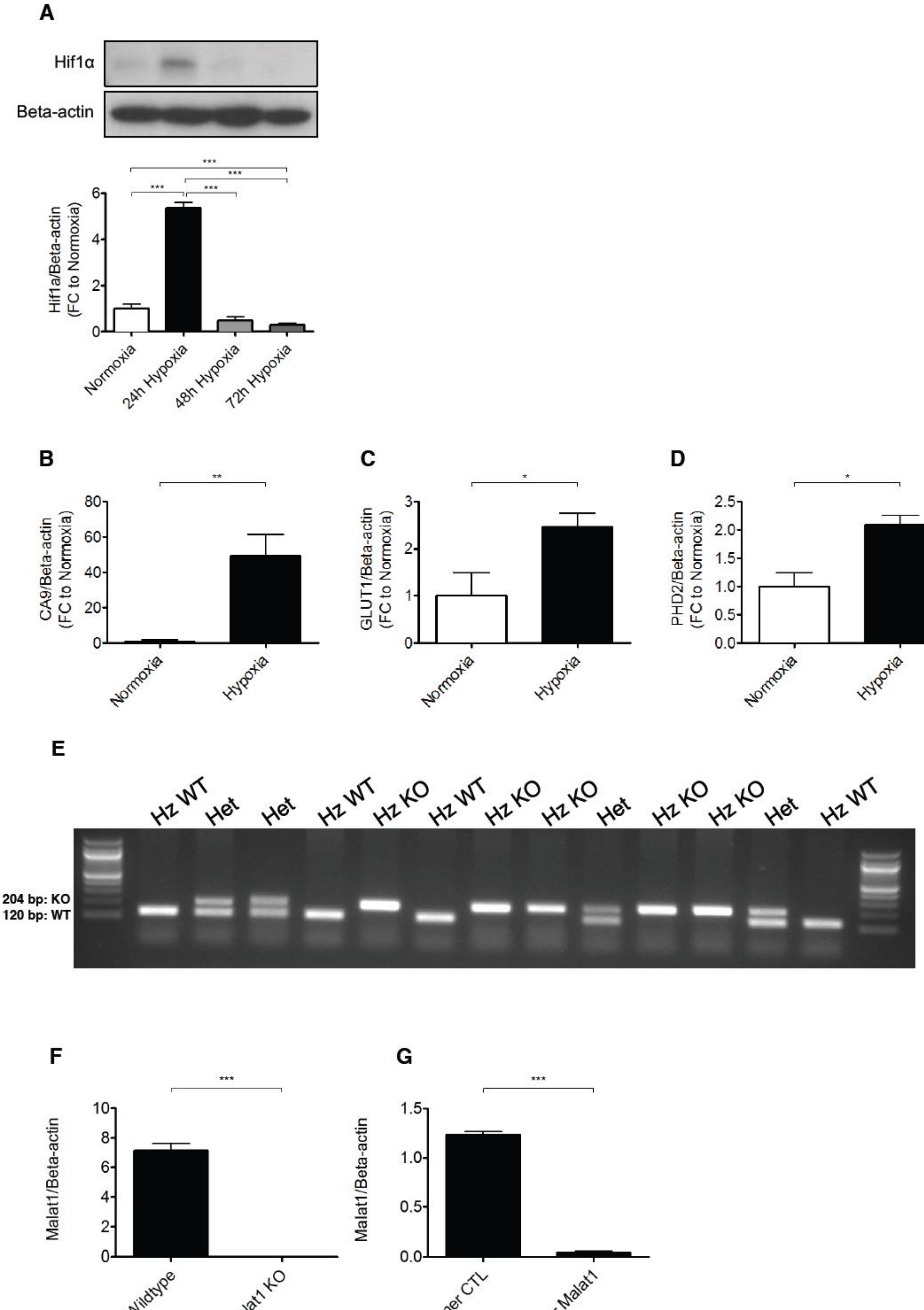

Supplemental Figure 2

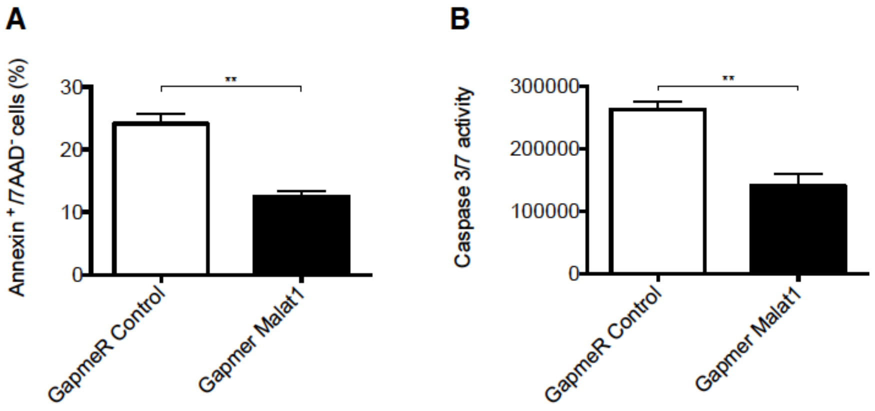

Supplemental Figure 3

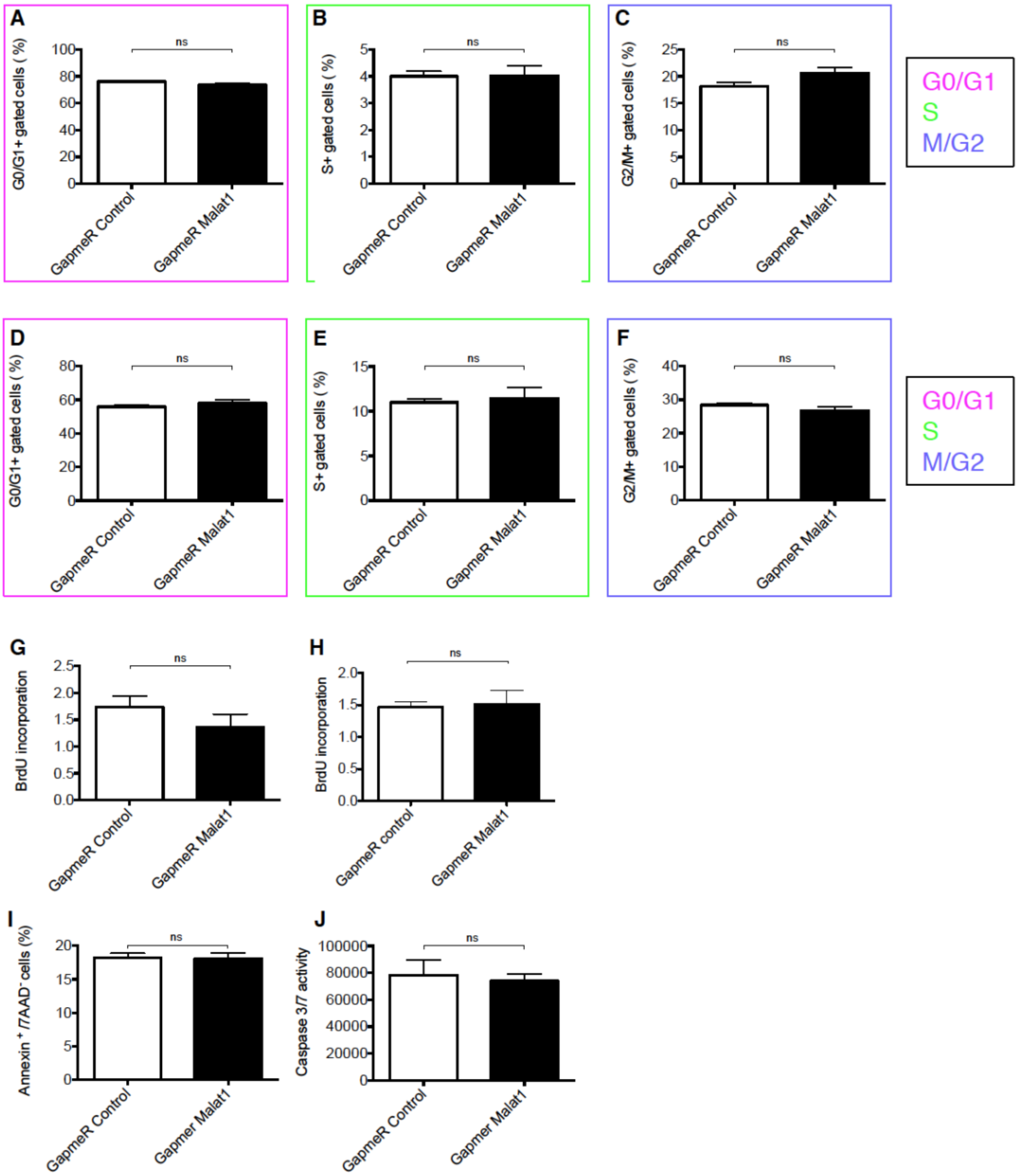

Supplemental Figure 4

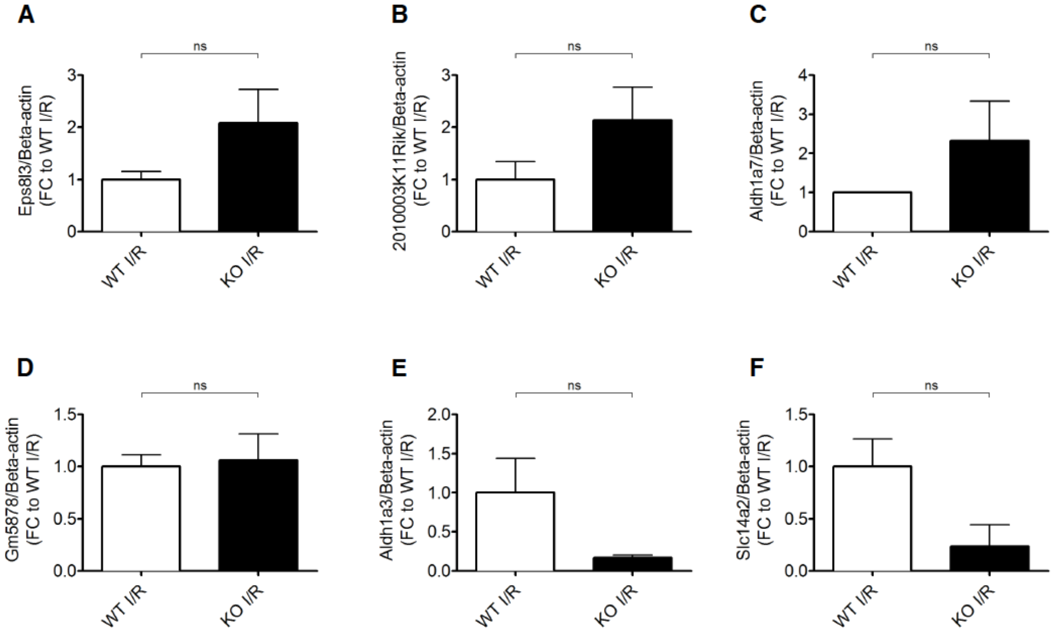

G

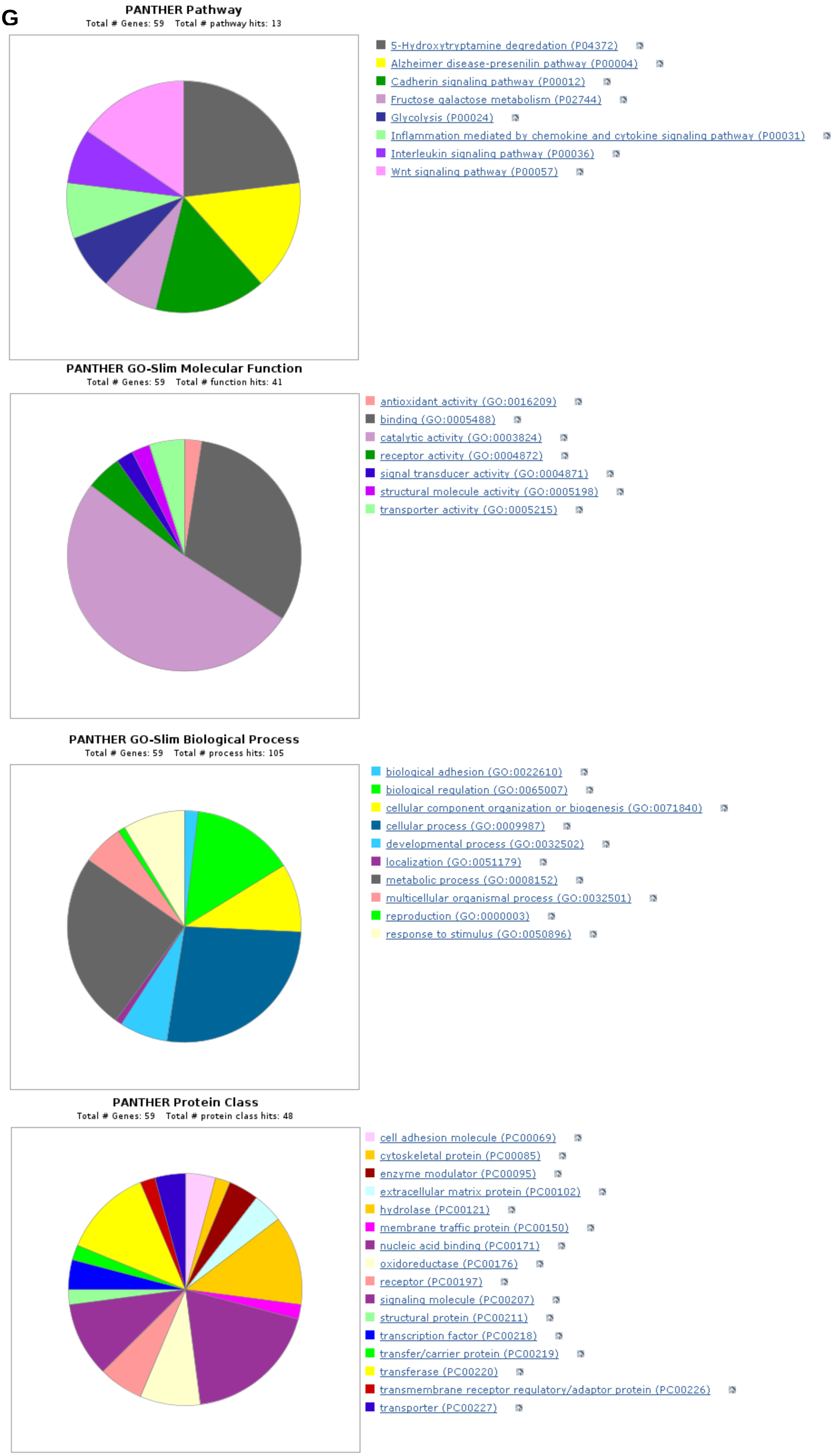
PANTHER Pathway
Total # Genes: 59 Total # pathway hits: 13
5-Hydroxytryptamine degredation (P04372)
Alzheimer disease-presenilin pathway (P00004)
Cadherin signaling pathway (P00012)
Fructose galactose metabolism (P02744)
Glycolysis (P00024)
Inflammation mediated by chemokine and cytokine signaling pathway (P00031)
Interleukin signaling pathway (P00036)
Wnt signaling pathway (P00057)
PANTHER GO-Slim Molecular Function
Total # Genes: 59 Total # function hits: 41
antioxidant activity (GO:0016209)
binding (GO:0005488)
catalytic activity (GO:0003824)
receptor activity (GO:0004872)
signal transducer activity (GO:0004871)
structural molecule activity (GO:0005198)
transporter activity (GO:0005215)
PANTHER GO-Slim Biological Process
Total # Genes: 59 Total # process hits: 105
biological adhesion (GO:0022610)
biological regulation (GO:0065007)
cellular component organization or biogenesis (GO:0071840)
cellular process (GO:0009987)
developmental process (GO:0032502)
localization (GO:0051179)
metabolic process (GO:0008152)
multicellular organismal process (GO:0032501)
reproduction (GO:0000003)
response to stimulus (GO:0050896)
PANTHER Protein Class
Total # Genes: 59 Total # protein class hits: 48
cell adhesion molecule (PC00069)
cytoskeletal protein (PC00085)
enzyme modulator (PC00095)
extracellular matrix protein (PC00102)
hydrolase (PC00121)
membrane traffic protein (PC00150)
nucleic acid binding (PC00171)
oxidoreductase (PC00176)
receptor (PC00197)
signaling molecule (PC00207)
structural protein (PC00211)
transcription factor (PC00218)
transfer/carrier protein (PC00219)
transferase (PC00220)
transmembrane receptor regulatory/adaptor protein (PC00226)
transporter (PC00227)

Blot and Gel images

HIF-1α Western blot

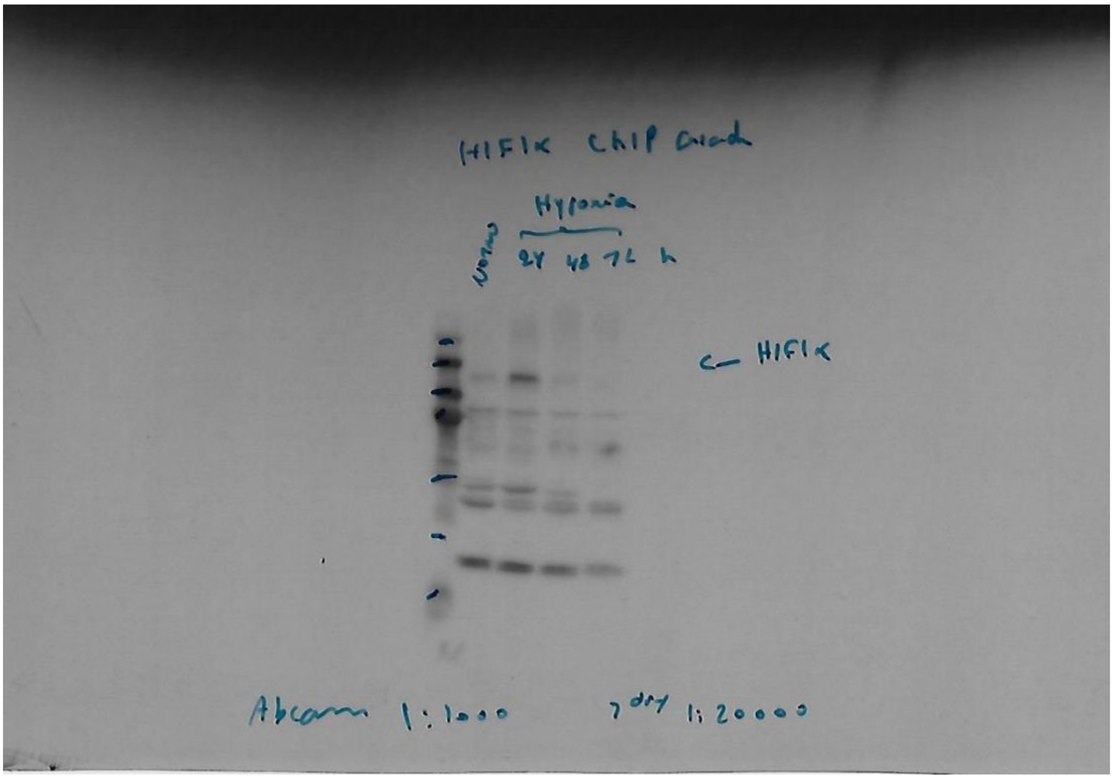

Beta-actin Western blot

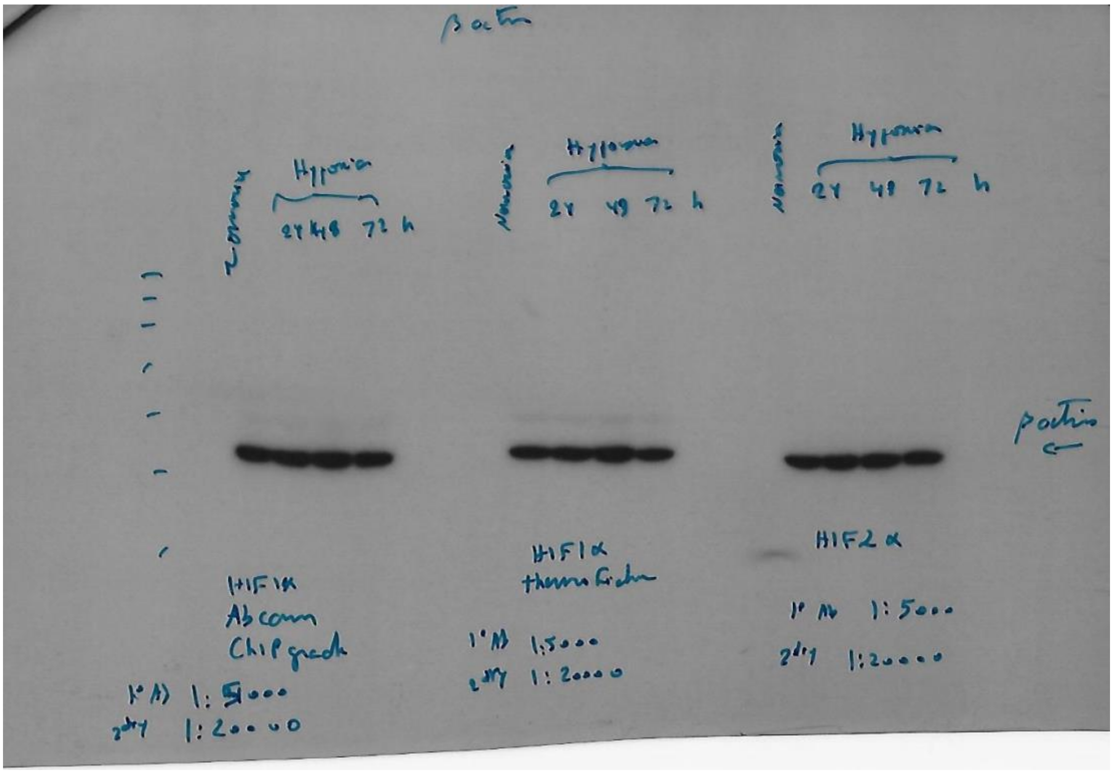

Genotyping of Malat1 knockout and wildtype mice

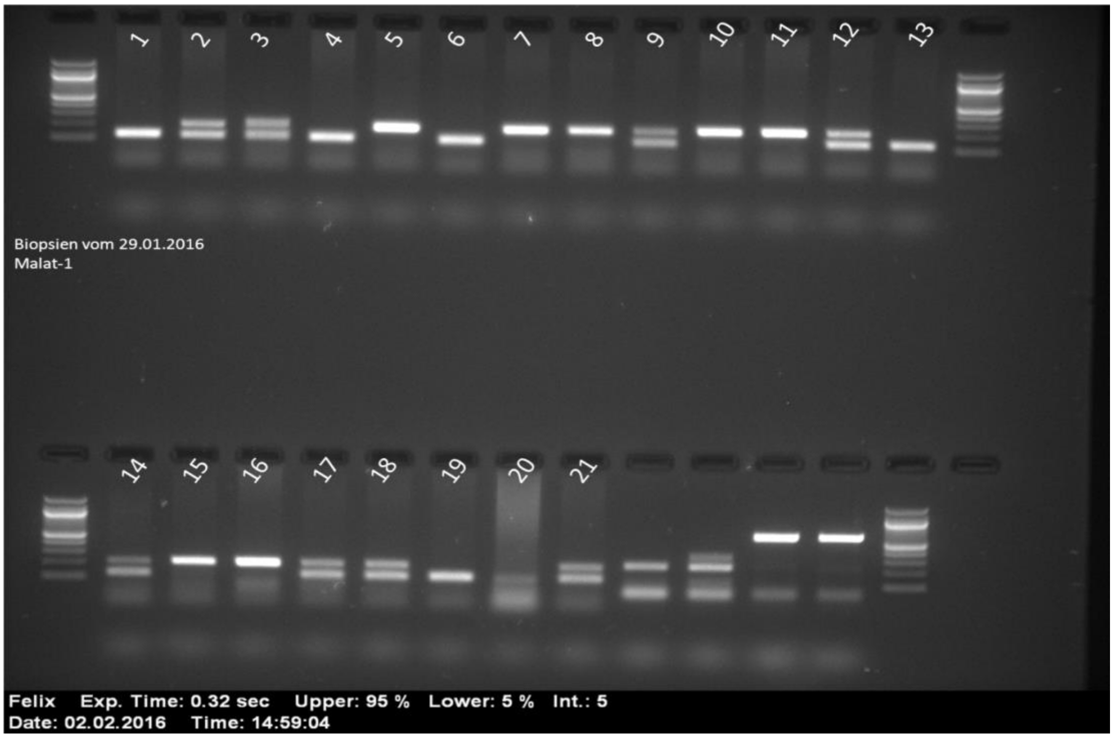

Supplement: Supplementary file 1 — Supplemental Materials and Methods [file 41598_2018_21720_MOESM1_ESM.pdf]
